# RHAMM drives formation of polyploid cancer cells and confers resistance to ER-targeted therapy in breast cancer

**DOI:** 10.1073/pnas.2535342123

**Published:** 2026-09-16

**Authors:** Shiyi Wu, Si Chen, Bohan Liu, Yuting Liu, Siyue Yang, Jiajie Hu, Yiqing He, Qinqing Liu, Yiwen Liu, Yan Du, Guoliang Zhang, Qian Guo, Feng Gao, Fen Tang, Yongming Xu, Cuixia Yang

**Affiliations:** ^a^ Department of Clinical Laboratory, Shanghai Sixth People’s Hospital Affiliated to Shanghai Jiao Tong University School of Medicine, Shanghai 200233, China; ^b^ Department of Molecular Biology, Shanghai Sixth People’s Hospital Affiliated to Shanghai Jiao Tong University School of Medicine, Shanghai 200233, China; ^c^ Faculty of Medical Laboratory Science, College of Health Science and Technology, Shanghai Jiao Tong University School of Medicine, Shanghai 200025, China; ^d^ Department of Breast Surgery, Shanghai Sixth People’s Hospital Affiliated to Shanghai Jiao Tong University School of Medicine, Shanghai 200233, China; ^e^ Department of Pain Management, Shanghai Sixth People’s Hospital Affiliated to Shanghai Jiao Tong University School of Medicine, Shanghai 200233, China

**Keywords:** RHAMM, YAP, polyploid cancer cell, polyploidization, endocrine resistance

## Abstract

Endocrine resistance in ER^+^ breast cancer remains a major clinical challenge. Here, we identify RHAMM as a key driver of resistance by orchestrating polyploid cancer cell (PCC) formation. Single-cell transcriptomics uncovered a G2/M-enriched, RHAMM^+^ subpopulation in endocrine-resistant tumors. Mechanistically, RHAMM binds Septin9/10 to promote aberrant cytoskeleton polymerization, activating YAP independent of Hippo signaling, which induces cytokinesis failure and facilitates PCC generation. Concurrently, RHAMM destabilizes p21 mRNA, enabling cell cycle progression despite genomic instability. The RHAMM–p21 axis serves as a bypass mechanism supporting polyploidization. Upon endocrine treatment, RHAMM is transcriptionally up-regulated by Slug. Clinically, RHAMM^high^ signatures are enriched in metastatic and recurrent ER^+^ tumors and correlate with poor prognosis, highlighting its therapeutic relevance. Importantly, targeting RHAMM or YAP abrogates PCC formation and restores fulvestrant sensitivity. These findings reveal RHAMM-mediated polyploidization as an adaptive mechanism underlying endocrine resistance, suggesting the therapeutic potential of targeting the RHAMM–YAP axis.

## Significance

Endocrine resistance in estrogen receptor-positive breast cancer remains a major clinical challenge. This study identifies a role for RHAMM in driving therapy failure through the induction of polyploidization—a process long associated with treatment resistance but poorly understood in this context. RHAMM binds Septin9/10 to activate YAP and destabilize p21, enabling cells to bypass cytokinesis and proliferate despite genomic instability. Clinically, high RHAMM expression correlates with metastasis and poor survival. Importantly, disrupting this axis abolishes polyploid cells and restores fulvestrant sensitivity, pinpointing an actionable vulnerability and providing a rationale for combination strategies to improve patient outcomes.

Estrogen receptor-positive (ER^+^) breast cancer comprises 80 to 90% of new diagnoses globally (1). While endocrine therapies—such as SERMs (selective estrogen receptor modulators, e.g., tamoxifen), ER degraders (e.g., fulvestrant), and aromatase inhibitors (e.g., anastrozole)—have significantly reduced recurrence and mortality, acquired resistance drives relapse in approximately 20% of patients, often through metastatic progression (2). Resistance mechanisms involve adaptive epigenetic alterations, dysregulated growth signaling, and tumor microenvironment interactions, collectively enabling ER-independent proliferation and treatment evasion. Deciphering these mechanistic drivers is crucial for developing novel targeted strategies to overcome resistance and improve long-term outcomes.

Whole-genome duplication (WGD) or polyploidization is a macroevolutionary event highly prevalent in cancer, occurring in approximately one-third of solid tumors (3) and over half of metastatic cases (4). Despite its association with poor prognosis and therapeutic resistance, WGD promotes genomic instability (CIN) and aneuploidy—features typically linked to cell vulnerability (5). This presents a central paradox: How do cancer cells exploit WGD to survive catastrophic genomic stress while evading the detrimental effects of polyploidy or aneuploidy? A key manifestation is the emergence of polyploid cancer cells (PCCs). These cells arise via cell fusion, endoreplication, or cytokinesis failure, and exhibit remarkable tolerance to chromosomal instability (6). Observed both in vitro and in vivo following chemotherapy or radiation, PCCs demonstrate adaptive mechanisms that fuels resistance and evolutionary plasticity (7). Their ability to mitigate aneuploidy-associated toxicity while promoting survival challenges conventional models of tumor evolution. Notably, in ER^+^ breast cancer, previous studies reported that WGD is linked to increased morbidity (3), yet its role in acquired endocrine resistance remains unclear. Understanding whether and how polyploidization contributes to resistant phenotypes in this context represents a critical unanswered mechanistic question.

The hyaluronan-mediated motility receptor (RHAMM/HMMR) localizes to both the cell surface and intracellular compartments, engaging hyaluronan, ERK, and microtubules to promote cell motility, signaling, and oncogenesis (8). Through centrosomal localization, it also plays a critical role in spindle integrity and regulates dynein-mediated processes during cell division (9). Although RHAMM is frequently overexpressed in carcinomas such as breast, prostate, and hematologic malignancies, and is linked to metastasis and poor prognosis, its oncogenic role is context dependent (10, 11). Paradoxically, RHAMM deletion accelerates tumorigenesis in pancreatic cancer models, and loss of function initiates seminoma development (12). While RHAMM has been implicated as a susceptibility gene in breast cancer (13), its potential involvement in endocrine therapy resistance remains unexplored.

Here, through unbiased scRNA-seq, we identify RHAMM as a defining feature of a G2/M-enriched subpopulation in fulvestrant-resistant murine breast tumors. We found that up-regulated RHAMM in resistant ER^+^ breast cancer cells not only positively correlates with the resistant phenotype but may also accelerate resistance evolution by promoting polyploidization/WGD processes; this finding provides an insight into the mechanisms underlying endocrine therapy failure.

## Results

### Single-Cell Profiling Identifies a G2/M-Enriched Cancer Cell Subpopulation Linked to Acquired Endocrine Resistance Characterized by RHAMM Overexpression.

**Fig. 1.**
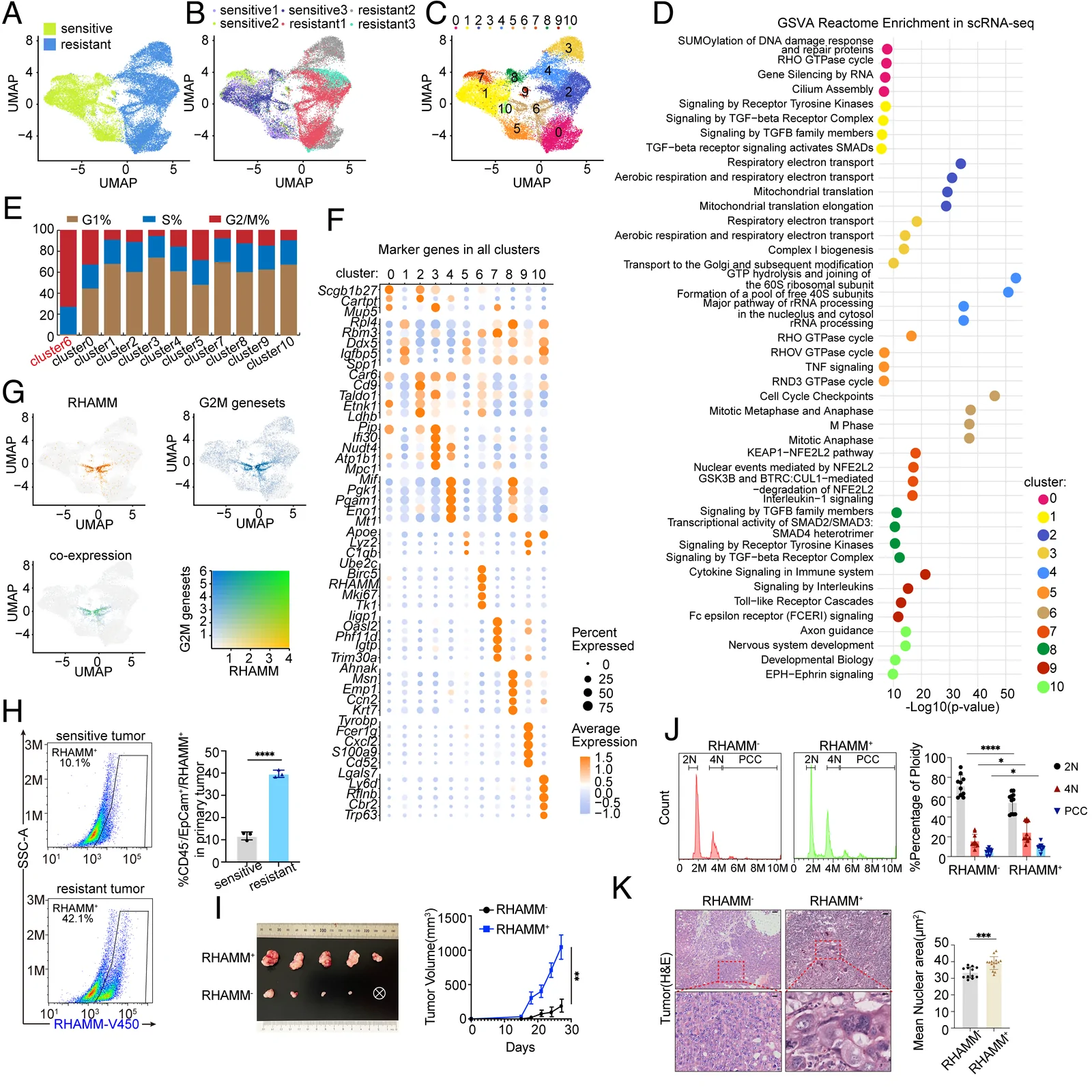
RHAMM contribute to endocrine resistance. (*A*) Uniform manifold approximation and projection (UMAP) plot showing the different cell populations in the BCs collected from fulvestrant-sensitive and -resistant mouse models. (*B*) UMAP plot showing sample contributions in the BCs from fulvestrant-sensitive and -resistant mouse models (n = 3 per group). (*C*) UMAP plot showing multiple cell clusters in the BCs collected from fulvestrant-sensitive and -resistant mouse models. (*D*) Dot plot showing top 4 reactome enrichment pathways of each cluster. (*E*) Proportion of cells in cell cycle phases per cluster was quantified by the Cyclone program. (*F*) Dot plot showing the representative markers for each cancer cell cluster. (*G*) Blended feature plots showing coexpression of *RHAMM* and G2M gene sets (*Mki67, Pttg1, Ccna2, Cdca3, Cdk1, Tpx2, Cenpa*). (*H*) FACS analysis of the proportion of RHAMM^+^ cancer cells in tumors from fulvestrant-sensitive and -resistant mouse models (n = 3 per group). (*I*) Tumor growth following orthotopic injection of RHAMM^−/+^ cancer cells sorted from resistant tumors, with fulvestrant treatment (n = 5/group). (*J*) Cell cycle and polyploidy analysis of FACS-sorted RHAMM^−/+^ cells from fulvestrant-resistant tumors were performed using propidium iodide (PI) DNA staining. (*K*) Representative H&E-stained images of PCCs and bar chart of mean nuclear areas in tumor sections from (*I*). (Scale bar, *Top*: 50 μm, *Bottom*: 10 μm.) Data were collected from a mean nuclear area of three sets of tumors. Data represent the mean (±SD). *P < 0.05, **P < 0.01, ****P < 0.0001.

To understand the mechanism of acquired resistance to endocrine therapy, we developed fulvestrant-resistant BC derived from MMTV-PyMT mouse models (14). Reanalysis of our scRNA-seq data of BC tumors from mouse models (14) revealed a clear segregation in epithelial cells between the two tissue groups, forming 11 unique cell subpopulations (Fig. 1 *A*–*C* and *SI Appendix*, Fig. S1*A*). Intriguingly, cluster 6 was identified as the only subpopulation shared between the two groups (Fig. 1*C*). Gene set variation analysis (GSVA) revealed that genes associated with cell cycle checkpoint and mitosis constituted the most up-regulated pathway in cluster 6 (Fig. 1*D* and Dataset S1). Moreover, cell cycle phase assignment using Cyclone indicated that cluster 6 contained a substantially higher proportion of cells in G2M phase compared to other clusters—73.32% vs, 5.84 to 33.05% (*SI Appendix*, Table S1 and Fig. 1*E*)—and exhibited elevated expression of key cell cycle regulators including *Ube2c, Tk1, Mki67, Cenpf, Cenpa, Rhamm, Cdk1, Cdc20, Ccna2, and Pttg1* (Fig. 1*F* and *SI Appendix*, Fig. S1*B*). Notably, RHAMM transcripts exhibited specific enrichment in G2/M-phase cells within cluster 6, showing clear colocalization with established G2/M marker genes (Fig. 1*G*). To further investigate this association, we stratified all tumor epithelial cells from the scRNA-seq dataset into RHAMM^+^ and RHAMM^−^ populations based on RHAMM expression levels. Subsequent pathway enrichment analysis revealed marked activation of mitosis-related pathways exclusively in RHAMM^+^ cells (*SI Appendix*, Fig. S1*C* and Datasets S2 and S3), providing compelling evidence for a strong association between RHAMM overexpression and the G2/M cell cycle phase.

We next sought to determine whether RHAMM-enriched subpopulations are associated with acquired therapy resistance. Notably, resistant tumors displayed a significantly expanded RHAMM^+^ population compared to therapy-sensitive controls (Fig. 1*H*). Concordantly, both resistant tumors and in vitro derived resistant breast cancer cells exhibited substantially up-regulated RHAMM expression (*SI Appendix*, Fig. S1 *D* and *E*), implying a potential functional link between RHAMM abundance and the resistant phenotype. Additionally, the “stem cell population maintenance” gene set was enriched in RHAMM^+^ resistant cells (*SI Appendix*, Fig. S1*F*), indicating a heightened stemness state in this population. To further elucidate the contribution of RHAMM^+^ cancer cells to endocrine therapy resistance, limiting dilution sphere formation assays were performed and revealed that RHAMM^+^ cancer cells exhibited a higher frequency of tumor-initiating cells (TICs) in response to fulvestrant treatment compared to RHAMM^-^ cells (*P* = 0.000134; *SI Appendix*, Fig. S1*G*). This indicates that RHAMM^+^ populations possess an intrinsic advantage in sustaining stemness and promoting tumor sphere formation under endocrine therapy pressure, underscoring their superior tumor-initiating potential of RHAMM^+^ luminal-like cancer cells compared to their RHAMM^−^ luminal-like cancer cells.

To further validate these findings in vivo, we orthotopically transplanted RHAMM^+^ or RHAMM^−^ cancer cells (sorted from resistant tumors) into mouse mammary fat pads. RHAMM^+^ tumors exhibited greater insensitivity to fulvestrant than RHAMM^−^ tumors (Fig. 1*I*), along with elevated expression of CD44 and Ki67 (*SI Appendix*, Fig. S2), indicative of enhanced proliferative capacity and stemness. To understand the properties of the RHAMM^+^ cells, we analyzed DNA content by propidium iodide staining. The results showed an increased proportion of cells with 4N or higher DNA content and a decrease in those with 2N among sorted RHAMM^+^ resistant cells (Fig. 1*J*), suggesting cell cycle dysregulation. Notably, we observed a marked increase in PCCs (defined as DNA content >4N) in the RHAMM^+^ population compared with the RHAMM^−^ population (Fig. 1*J*), implicating polyploidization in this resistant subset. Consistent with this finding, resistant cancer cells exhibited a significantly higher number of PCCs—approximately twofold enriched—compared to their sensitive counterparts (*SI Appendix*, Fig. S1 *H* and *I*), further supporting the association between polyploidization and treatment resistance. Moreover, RHAMM^+^ tumors displayed enlarged nuclei (Fig. 1*K*), consistent with enhanced polyploid features. Given recent evidence that PCCs retain proliferative capacity (15), our results highlight a causal role of RHAMM^+^ cancer cells in promoting polyploidization and endocrine resistance, which is likely to confer a worse clinical outcome in ER^+^ BC patients.

### High Expression of RHAMM Is Associated with WGD in ER^+^ BC.

**Fig. 2.**
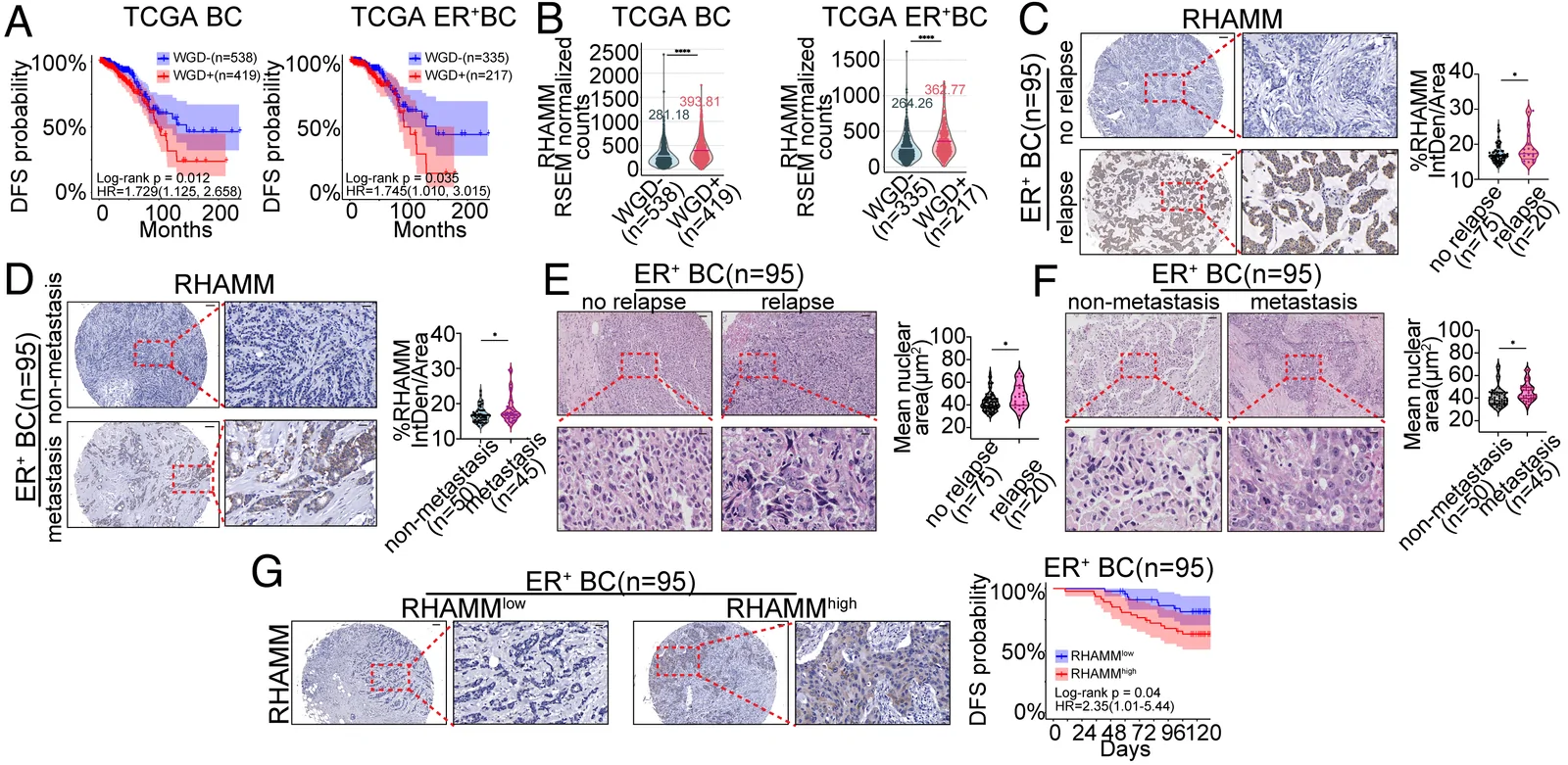
High expression of RHAMM is associated with unfavorable treatment outcomes and WGD in ER^+^ BC patients. (*A*) Survival probability analysis of WGD^−^ vs. WGD^+^ breast tumors in the TCGA across molecular subtypes or in ER^+^ subtypes. (*B*) Violin plot depicting per-sample mean gene expression of RHAMM in WGD^−^ and WGD^+^ BC (overall) or ER^+^ BC samples from TCGA data. Significance calculated using the two-sided Wilcoxon rank-sum test is included. RSEM: RNA-Seq by expectation–maximization. The dotted line represents the mean. (*C*) Representative images and quantification of RHAMM frequencies in primary ER^+^ BC samples from patients who experienced relapse (n = 20) vs. those who did not (n = 75). The dotted lines show the median and quartile values. (Scale bar, *Top*: 100 μm, *Bottom*: 20 μm.) (*D*) Representative images and quantification of RHAMM frequencies in primary ER^+^ BC samples from patients with metastasis (n = 45) or without (n = 50). The dotted lines show the median and quartile values. (Scale bar, *Top*: 100 μm, *Bottom*: 20μm.) (*E* and *F*) Representative images and quantification of nuclear area in primary ER^+^ BC samples from patients with relapse (n = 20) vs. those without (n = 75) (*E*), and from patients with metastasis (n = 45) vs. those without metastasis (n = 50) (*F*). The dotted lines show the median and quartile values. (Scale bar, *Top*: 50 μm, *Bottom*: 10 μm.) (*G*) Representative images and survival probability analysis (DFS) in ER^+^ breast cancer patients stratified by high (n = 47) vs. low (n = 48) RHAMM expression. (Scale bar, *Top*: 100 μm, *Bottom*: 20 μm.) Data represent the mean (±SD). *P < 0.05.

To investigate whether RHAMM contributes to endocrine resistance via polyploidization/WGD—a mechanism linked to increased evolvability and treatment failure—we analyzed its association with WGD in breast cancer. Using ABSOLUTE algorithm to assess polyploidy/WGD status in human breast cancers from TCGA, we stratified tumors based on whether they had experienced a WGD event. This cohort comprised patients with advanced/metastatic disease who underwent prospective clinical sequencing. WGD was significantly associated with reduced overall survival in the full BC cohort (HR = 1.729, 95% CI: 1.125 to 2.658, *P* = 0.012; Fig. 2 *A*, *Left*) and in ER^+^ breast cancer (HR = 1.745, 95% CI: 1.010 to 3.015, *P* = 0.035; Fig. 2 *A*, *Right*). Notably, RHAMM expression was elevated in WGD^+^ tumors both overall and in ER^+^ subtypes (Fig. 2*B*). Furthermore, to determine the clinical implications of RHAMM in breast cancer progression, by using immunohistochemistry on a tissue microarray comprising 134 breast tumor samples across molecular subtypes (*SI Appendix*, Table S2). Specimens were obtained from patients with or without metastasis at diagnosis or recurrence. In all breast cancers across molecular subtypes or ER^+^ subtypes, RHAMM expression and polyploidization (cells characterized by enlarged cell sizes with swelling mono- or multinuclei) were significantly elevated in metastatic and recurrent cases compared to nonmetastatic and recurrence-free samples (Fig. 2 *C*–*F* and *SI Appendix*, Fig. S3 *A* and *B*). When samples were classified into RHAMM^high^ and RHAMM^low^ groups based on median IHC scores, patients with high RHAMM expression had significantly shorter disease-free survival (DFS) (Fig. 2*G* and *SI Appendix*, Fig. S3*C*). These findings further suggest that high expression of RHAMM is associated with unfavorable treatment outcomes.

### RHAMM Promotes the Generation of PCCs.

**Fig. 3.**
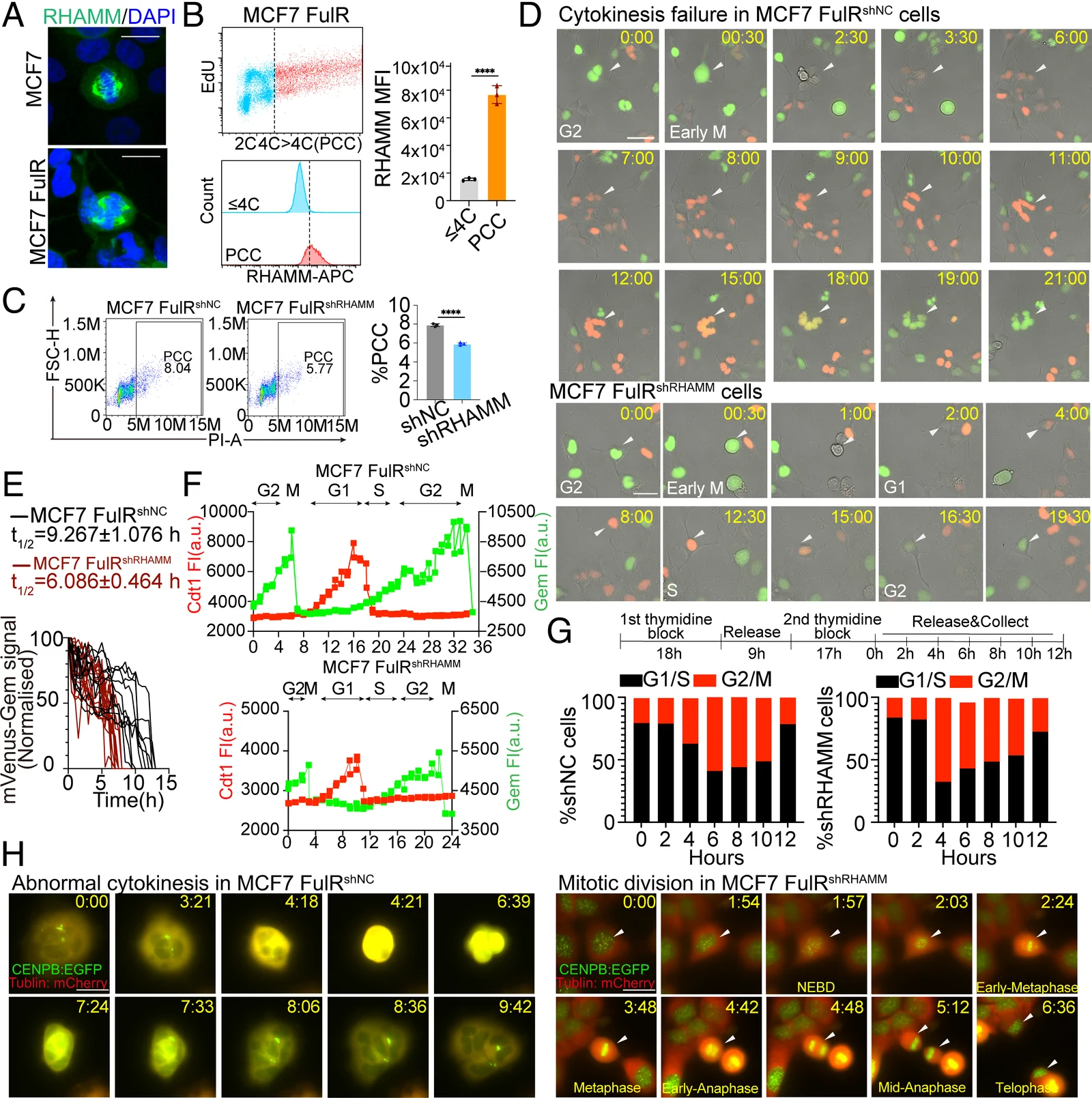
RHAMM^+^ cancer cells generate PCCs. (*A*) Immunofluorescence staining showing RHAMM expression and localization in MCF7 and MCF7 FulR cells. (Scale bars, 50 μm.) (*B*) Analysis of mean fluorescence intensities (MFI) of RHAMM between ≤4N and PCC in MCF7 FulR cells distinguished by Edu/Hoechst staining-based FACS. (*C*) Changes in PCCs proportion were analyzed by PI staining in MCF7 FulR^shNC^ and MCF7 FulR^shRHAMM^ cells. (*D*) Time-lapse imaging of MCF7 FulR^shNC^ and MCF7 FulR^shRHAMM^ cells labeled with FUCCI. FUCCI and phase contrast images were merged. The FUCCI system labels nuclei in red at the G1 phase, yellow at the G1/S transition phase, and green at the S/G2/M phases. Representative images showing MCF7 FulR^shNC/shRHAMM^ cells in distinct cell cycle phase. (Scale bars, 50 μm.) (*E*) Normalized time-course degradation of mVenus-Gem in live MCF7 FulR^shNC/shRHAMM^ cells. Each line represents a single-cell tracking (n = 20 per condition). Mean half-life (t_1/2_ ± SD) is shown. (*F*) Temporal profiles of fluorescence intensities (FI) of mCherry-Cdt1 and mVenus-Gem of cells in MCF7 FulR^shNC/shRHAMM^ cells. a.u., arbitrary unit. (*G*) Schematic representation of double thymidine block for cell synchronization in early S-phase is shown. Cell synchronization was monitored by flow cytometry of PI-stained cells. The percentage of cells in G1/S or G2/M is plotted. (*H*) Representative time-lapse images of MCF7 FulR^shNC/shRHAMM^ cells coexpressing CENPB:EGFP (green) and Tubulin:mCherry (red) undergoing a mitotic division. Nuclear envelope breakdown, NEBD. Data represent the mean (±SD). ****P < 0.0001.

Since RHAMM has been implicated in centrosome abnormalities (16, 17), we investigated whether RHAMM contributes to fulvestrant-induced accumulation of PCCs. Intriguingly, immunofluorescence analysis revealed specific RHAMM localization at the spindle apparatus, along with a pronounced presence of multipolar spindles in fulvestrant-resistant MCF7 FulR cells (Fig. 3*A*). EdU incorporation assays further demonstrated that RHAMM levels were significantly elevated in the PCC (>4C) population compared to the ≤4C population (Fig. 3*B*). Based on these findings, we next investigated whether RHAMM hyperaccumulation contributes to the increased polyploidy. Single RHAMM^+^ BC cell was isolated from resistant BC cells by FACS sorting, and then the proportion of PCCs in RHAMM^+^-derived daughter cells was found significantly higher compared to RHAMM^−^ BC cells (*SI Appendix*, Fig. S4*A*). Indeed, the morphological features of PCCs observed under light microscopy were consistent with the flow cytometry data (*SI Appendix*, Fig. S4*B*). Interestingly, RHAMM^+^-derived daughter cells still preserved its high level of RHAMM expression and resistance to fulvestrant treatment (*SI Appendix*, Fig. S4 *C* and *D*). Moreover, high RHAMM expression and fulvestrant resistance persisted even after 7 or 14 d of drug withdrawal (*SI Appendix*, Fig. S4 *E* and *F*). Furthermore, the knockdown of RHAMM using lentiviral short hairpin RNA (shRNA) in resistant cancer cells resulted in decreased polyploidy (Fig. 3*C*), suggesting a role of RHAMM in the generation of PCCs. Using FACS, we isolated pure diploid and polyploid (PCC) populations from MCF7 FulR^shNC/shRHAMM^ cells and assessed their colony formation and proliferative capacity under fulvestrant treatment. In control (shNC) cells, PCCs displayed significantly enhanced colony formation and greater resistance to fulvestrant compared with their diploid counterparts (*SI Appendix*, Fig. S4 *G* and *H*). Notably, RHAMM knockdown not only diminished fulvestrant resistance in both populations but also abolished the functional advantage of PCCs, resulting in no significant differences in colony formation or drug sensitivity between diploid cells and PCCs (*SI Appendix*, Fig. S4 *G* and *H*).

To determine how PCC was generated following RHAMM expression, we used fluorescent ubiquitination-based cell-cycle indicator (FUCCI) live-cell imaging (18). In cells expressing fluorescently tagged, truncated versions of Cdt1 and geminin, one can distinguish G1 (high mCherry-Cdt1), S (high mCherry-Cdt1 and high mVenus-Gem), and G2 (high mVenus-Gem) phases of the cell cycle (19). As shown, MCF7 FulR^shNC^ cells frequently exhibited increased size, multinucleation, and apparent initiation but noncompletion of mitosis, prolonged G2/M and G1 phase (green/no fluorescence/red; white arrow; Fig. 3 *D* and *F* and Movies S1–S4), generated daughter cells through bipolar or multipolar division. In contrast to the slow mVenus-Gem degradation in mitosis of MCF7 FulR^shNC^ cells (Fig. 3*E*), mVenus-Gem degradation was more rapid in MCF7 FulR^shRHAMM^: the half-time (t_1/2_) of degradation was 6.086 ± 0.464 h (median = 6.086 h), roughly 1.5 times shorter than MCF7 FulR^shNC^ cells (9.267 ± 1.076 h). Although direct inhibitors of RHAMM are not currently available, we investigated 4-methylumbelliferone (4-MU), which has been reported to suppress RHAMM expression (20). Consistent with these reports, we confirmed that 4-MU treatment reduced RHAMM expression in MCF7 FulR cells (*SI Appendix*, Fig. S4 *I* and *J*). Moreover, PI staining and FUCCI analysis after 4-MU treatment recapitulated the effects as observed in MCF7 FulR^shRHAMM^—specifically, a reduction in PCCs and dysregulated cell cycle progression (*SI Appendix*, Fig. S4 *K* and *L*). Together, these results strongly support a significant role for RHAMM in promoting PCC formation.

To investigate if RHAMM expression influences cell cycle progression, cells were synchronized at early S-phase using a double thymidine block upon fulvestrant treatment. In control-resistant cells, progression into S phase occurred within 0 to 2 h, followed by entry into G2 phase at 2 to 4 h and mitosis at 4 to 12 h. In contrast, RHAMM depletion significantly accelerated S-phase reentry after release. Notably, 4 h after the second release, 67.0% of MCF7 FulR^shRHAMM^ cells had progressed into G2/M phase, compared to only 36.2% of control cells (Fig. 3*G*).

To visualize the dynamic changes of chromosomes and the spindle movement in resistant BC cells during the division process, we transduced MCF7 FulR cells with Cenpb-EGFP (kinetochore marker) and Tubulin-mCherry(microtubule marker). Results showed that PCCs found in MCF7 FulR^shNC^ underwent abnormal mitosis since it divided into three multinucleated daughter cells (Fig. 3*H* and Movie S5). By contrast, more cells in MCF7 FulR^shRHAMM^ divided via canonical mitosis (Fig. 3*H* and Movie S6). These findings suggest that RHAMM knockdown may mitigate the severe replication stress and genomic instability associated with fulvestrant-resistant cells.

### YAP Activation Drives Polyploidization in Endocrine-Resistant Cancer Cells.

**Fig. 4.**
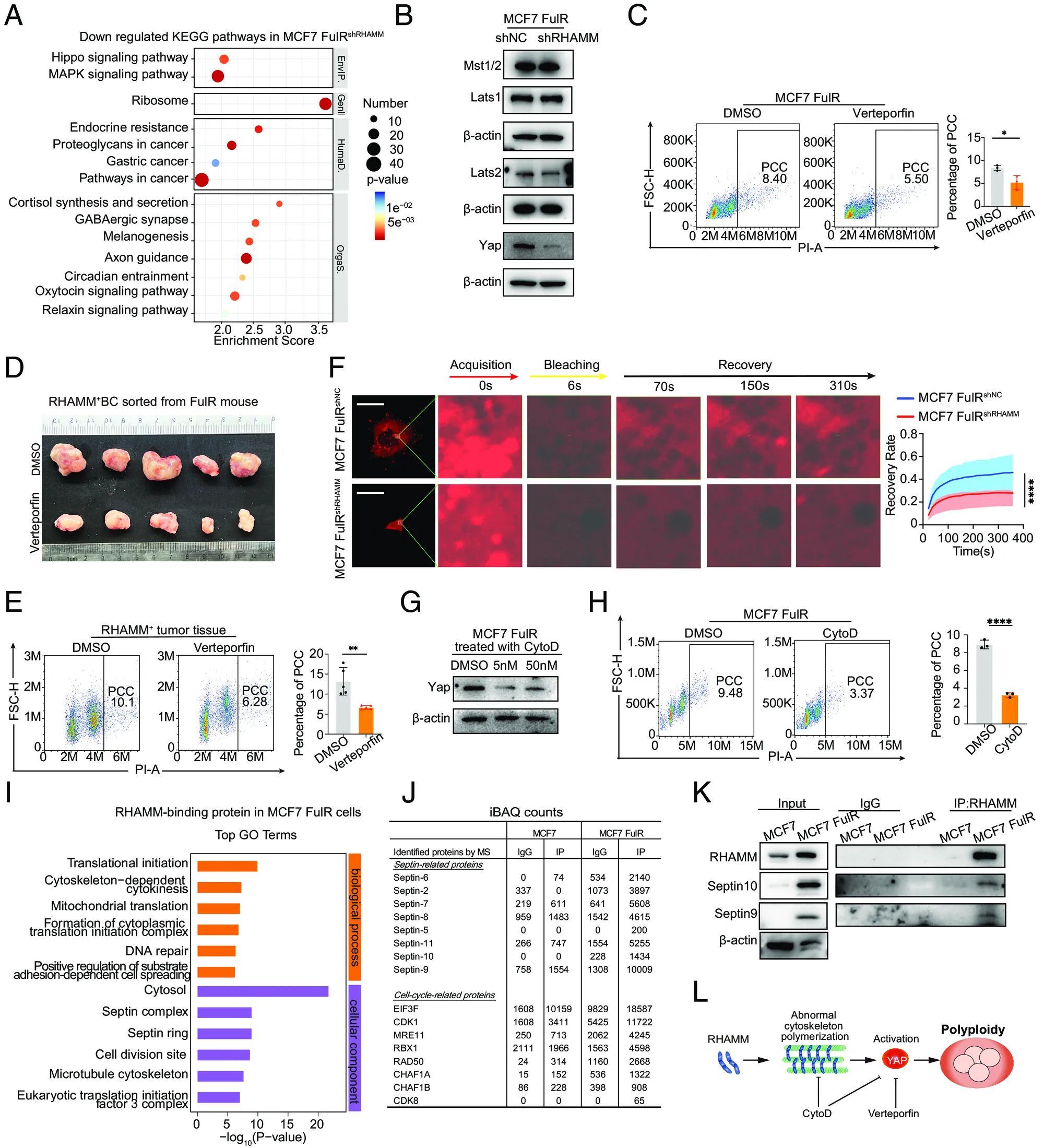
RHAMM activates YAP signaling to convert cancer cells into PCCs. (*A*) KEGG enrichments of differentially expressed genes that were down-regulated in MCF7 FulR^shRHAMM^ cells based on RNA-seq. (*B*) Western blot analysis of Hippo pathway-related protein expression in MCF7 FulR^shRHAMM^ cells. (*C*) PI staining-based analysis of the proportion of PCCs in MCF7 FulR cells after exposure to 5 μM verteporfin for 24 h. (*D*) RHAMM^+^ tumor cells derived from fulvestrant-resistant breast cancers were orthotopically implanted into Fvb mice. Once tumors reached a volume of 100 mm^3^, verteporfin (100 mg/kg) was injected intraperitoneally (i.p.) every other day and fulvestrant (50 mg/kg, i.m.) once a week. Representative images of the developed orthotopic transplants. (*E*) Cells isolated from (*D*) were subjected to cell cycle phase distribution and polyploidy analysis using PI staining. (*F*) Representative fluorescence recovery after photobleaching (FRAP) images at indicated time points of MCF7 FulR^shNC/shRHAMM^ cells. Statistical results of FRAP assays are shown on the left curves. (Scale bars, 50 μm.) (*G*) Western blot analysis of YAP expression in MCF7 FulR following treatment with cytochalasin D (CytoD) for 24 h. (*H*) PI staining-based analysis of changes in the proportion of PCC in MCF7 FulR cells following treatment with CytoD for 24 h. (*I*) GO analysis of RHAMM-interacting peptides identified by coimmunoprecipitation-based mass spectrometry assay (co-IP/MS) in MCF7 FulR cells. (*J*) The graph shows identified RHAMM interactors related to septin organization and the cell cycle in MCF7 and MCF7 FulR cells. iBAQ, intensity-based absolute quantification. (*K*) Interaction among RHAMM, septin 9, and septin 10 was analyzed by coimmunoprecipitation assays in MCF7 and MCF7 FulR. (*L*) A proposed working model for RHAMM binding to septins and thus induce abnormal actin polymerization to activate YAP signaling to enhance the formation of PCCs. Data represent the mean (±SD). *P < 0.05, **P < 0.01, ****P < 0.0001.

We sought to elucidate the mechanism through which RHAMM promotes the formation of PCCs. To determine how RHAMM influences transcriptional programs conducive to PCC formation, we performed RNA-seq analysis of MCF7 FulR^shRHAMM^ cells. Using a threshold of |log2FC| > 1 and FDR < 0.01, we found that RHAMM silencing significantly down-regulated 367 genes and up-regulated 336 genes (*SI Appendix*, Fig. S5*A*). Subsequent KEGG pathway analysis of these differentially expressed genes revealed a significant enrichment of genes associated with the negative regulation of the Hippo signaling pathway and MAPK signaling pathway (Fig. 4*A* and Dataset S4).

Based on this observation, we investigated whether RHAMM modulates Hippo or MAPK signaling pathway upon endocrine therapy. Immunoblotting after RHAMM knockdown revealed that, among all tested upstream regulators in both pathways, only RAS and YAP were significantly down-regulated (*SI Appendix*, Fig. S5 *B* and *C* and Fig. 4*B*). To determine which of these two acts as the functional effector, we used pharmacological inhibition. RAS inhibition with Salirasib (21, 22) did not affect PCC formation in resistant cells (*SI Appendix*, Fig. S5*D*), arguing against a direct functional role. In contrast, YAP inhibition using verteporfin [a potent YAP inhibitor (23)] markedly reduced the PCC population (Fig. 4*C*), indicating that YAP activity is essential for polyploidization. These results indicate that the Hippo/YAP axis, rather than RAS, is the key downstream pathway of RHAMM. Supporting this, YAP expression was significantly up-regulated in fulvestrant-resistant cells and tumors compared with their sensitive counterparts (*SI Appendix*, Fig. S5 *C*, *E*, and *F*). Given that YAP drives the diploid-to-polyploid transition and sustains polyploid cell proliferation (24), we hypothesized that RHAMM promotes PCC formation primarily through YAP modulation. Notably, RHAMM knockdown reduced YAP without altering LATS1/2 levels (Fig. 4*B*), pointing to a noncanonical, LATS-independent mechanism.

To assess the functional relevance of YAP in vivo, we injected RHAMM^+^ cancer cells isolated from fulvestrant-resistant tumors into the mammary fat pads of wild-type FVB mice. Concurrent administration of verteporfin and fulvestrant significantly suppressed tumor growth compared to the control group (Fig. 4*D* and *SI Appendix*, Fig. S5*G*), indicating that YAP inhibition restores fulvestrant sensitivity. Importantly, verteporfin-treated tumors exhibited a pronounced reduction in PCC frequency (Fig. 4*E*), further supporting a relevant mechanism through which RHAMM regulates YAP to drive PCC generation. Taken together, these results support a mechanism in which RHAMM drives PCC generation through activation of YAP-dependent transcriptional programs.

### RHAMM Promotes Abnormal Cytoskeleton Polymerization-Mediated Hippo-YAP Signaling to Drive PCC Formation.

Since the Hippo pathway effector YAP is known to be mechanosensitive and regulated by F-actin tension and polymerization (25). Accordingly, we found that RHAMM knockdown disrupted F-actin network architecture in fulvestrant-resistant cells (*SI Appendix*, Fig. S5*H*). To directly assess actin dynamics, we employed FRAP in cells expressing mCherry-actin—where the rate of fluorescence recovery serves as a proxy for actin polymerization (26). RHAMM depletion significantly reduced the rate of fluorescence recovery, indicating impaired actin polymerization (Fig. 4*F*). We next asked whether actin polymerization influences YAP expression and PCC formation. To test this, we induced actin depolymerization using cytochalasin D (CytoD) and observed a substantial decrease in YAP protein levels (Fig. 4*G* and *SI Appendix*, Fig. S5 *I* and *J*). Importantly, CytoD treatment also significantly reduced the proportion of PCCs (Fig. 4*H*). These results imply that RHAMM-mediated actin polymerization is essential for maintaining YAP stability and, consequently, for promoting polyploidization in response to endocrine treatment. Together, these findings suggest RHAMM as a key upstream regulator of actin cytoskeleton-driven Hippo-YAP signaling during the generation of PCCs. To elucidate the molecular mechanisms through which RHAMM promotes actin polymerization, we sought to identify its functional binding partners by coimmunoprecipitation coupled with mass spectrometry (IP/MS). Gene Ontology (GO) analysis of RHAMM-interacting peptides revealed significant enrichment of proteins involved in microtubule cytoskeleton, septin ring, cytoskeleton-dependent cytokinesis, and the microtubule cytoskeleton (Fig. 4*I*). This result highlighted these processes as central to the RHAMM-associated protein complex, suggesting a role for RHAMM in regulating cytoskeletal dynamics during mitotic progression.

Notably, among the candidate interactors identified by MS were multiple proteins implicated in cell cycle regulation and members of the septin family (Fig. 4*J*), pointing to a potential mechanism by which RHAMM influences cytokinesis and genome duplication. To further validate these interactions, we performed co-IP experiments in MCF7 FulR cells and confirmed a specific enhancement of the binding between RHAMM and Septin9/10 (Fig. 4*K* and *SI Appendix*, Fig. S5*K*). Supporting the functional relevance of this association, immunofluorescence analysis further demonstrated markedly increased colocalization of RHAMM with both Septin10 and Septin9 in fulvestrant-resistant MCF7 and T47D cells compared to their sensitive counterparts (*SI Appendix*, Fig. S5 *L* and *M*). Given that septin family proteins serve as core structural components that interface with actin filaments, microtubules, and are indispensable for cytokinesis (27), the identified interaction between RHAMM and septins thus implies a mechanism whereby RHAMM promotes polyploidization by directly engaging with septin complexes, thereby impairing cytokinesis and promoting genomic duplication, ultimately fostering the emergence of PCCs (Fig. 4*L*).

### RHAMM-Mediated Reduction of p21 mRNA Stability Promotes Polyploidization.

**Fig. 5.**
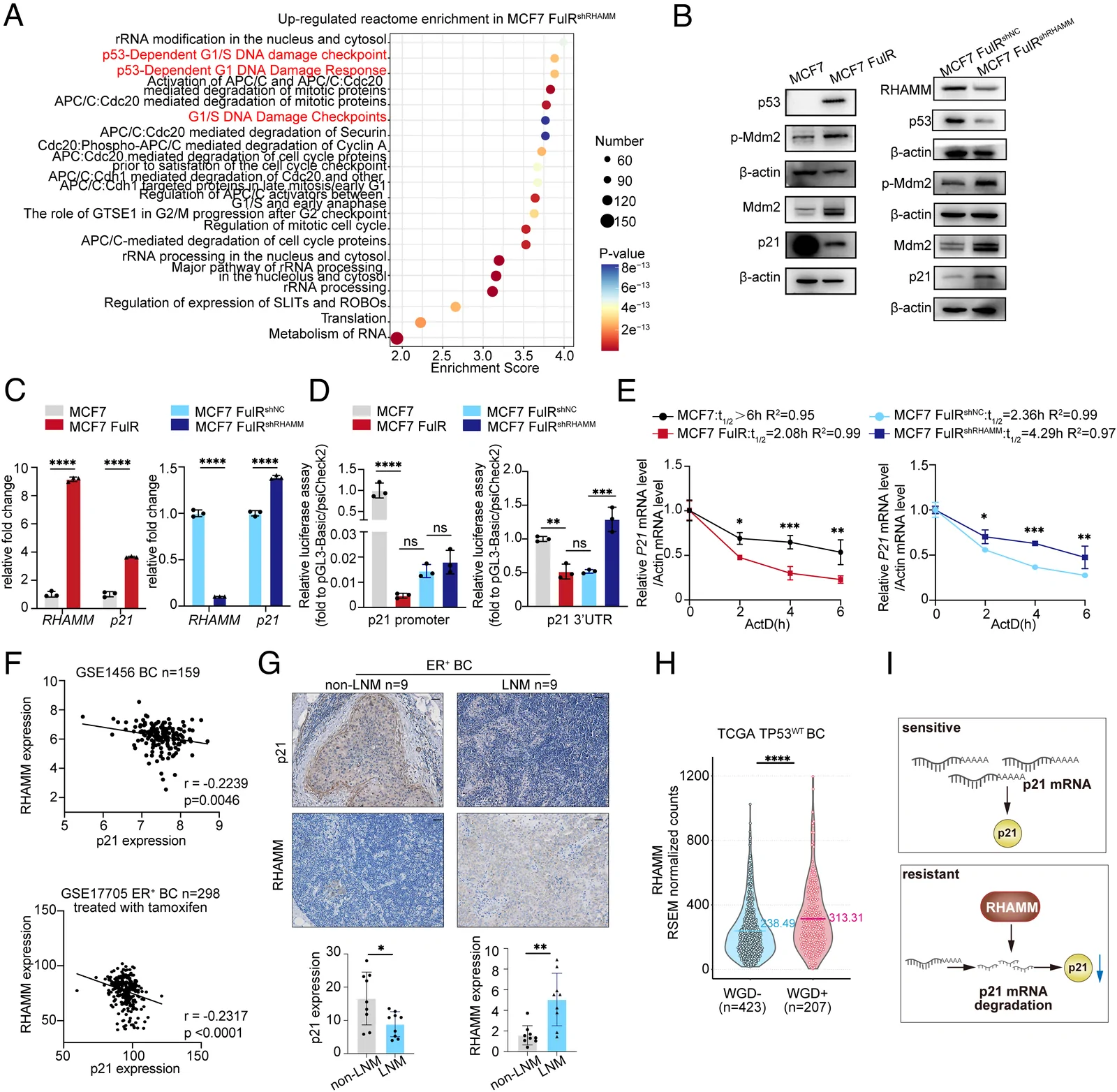
RHAMM promotes PCCs survival via p21 signaling inhibition. (*A*) Reactome enrichments of differentially expressed genes that were up-regulated in MCF7 FulR^shRHAMM^ cells based on RNA-seq. (*B*) Western blot analysis of p53, p21, Mdm2, and p-Mdm2 (phosphorylated-Mdm2) expression in MCF7/MCF7 FulR cells and MCF7 FulR^shNC/shRHAMM^ cells. (*C*) RT-qPCR analysis of p21 and RHAMM mRNA levels in MCF7/MCF7 FulR cells and MCF7 FulR^shNC/shRHAMM^ cells. (D) The relative luciferase activity of p21 promoter and 3′-UTR in MCF7/MCF7 FulR cells and MCF7 FulR^shNC/shRHAMM^ cells. (*E*) Analysis of p21 mRNA level in MCF7/MCF7 FulR and MCF7 FulR^shNC/shRHAMM^ cells following treatment with 5 μg/mL of actinomycin D (Act D) at different time points. (*F*) Negative correlation between p21 and RHAMM expression in human breast cancers from GEO database (GSE1456: n = 159, GSE17705: n = 298). (*G*) Representative images of p21 and RHAMM expression in human ER^+^BC samples with lymph node metastasis (LNM, n = 9) and without (non-LNM, n = 9). (Scale bars, 50 μm.) (*H*) Violin plot depicting per-sample mean gene expression of RHAMM in WGD^−^ (n = 423) and WGD^+^ (n = 207) TP53^WT^ BC samples. RSEM: RNA-Seq by expectation–maximization. The dotted line represents the mean. (*I*) A proposed working model for RHAMM down-regulates p21 mRNA stability. Data represent the mean (±SD). Data represent the mean (±SD). ns (not significant), *P < 0.05, **P < 0.01, ***P < 0.001, ****P < 0.0001.

Previous studies suggest that polyploid cells must overcome the G1 restriction point to reenter the cell cycle following cytokinesis failure, though the existence of a p53-dependent “tetraploidy checkpoint” remains controversial (28, 29). To understand how RHAMM promotes PCC formation, we reanalyzed the transcriptional profile of RHAMM-knockdown cells. Pathway enrichment analysis revealed a significant shift in genes associated with the p53-mediated G1/S DNA damage checkpoint (Fig. 5*A*), suggesting that RHAMM may modulate cell cycle machinery to facilitate polyploidization.

We next examined key components of the p53 pathway by immunoblotting. Compared with parental MCF7 cells, MCF7 FulR cells exhibited elevated p53 and Mdm2 protein levels but reduced p21 expression (Fig. 5*B*). Strikingly, RHAMM knockdown in resistant cells further increased Mdm2 and p21 expression, while decreasing p53 expression (Fig. 5*B* and *SI Appendix*, Fig. S6*A*). ChIP-PCR confirmed reduced p53 occupancy at the p21 promoter upon RHAMM silencing (*SI Appendix*, Fig. S6*B*). Therefore, p21 expression increased despite diminished p53 binding and protein levels—a finding that contrasts with the RNA-seq enrichment of p53 dependent G1/S pathways. Subsequent analysis revealed that MCF7 FulR cells exhibited elevated p21 mRNA levels but reduced p21 protein levels compared with parental MCF7 cells (Fig. 5 *B* and *C*), indicating posttranscriptional repression of p21 in resistant cells. Notably, RHAMM knockdown in MCF7 FulR cells increased both p21 mRNA and protein (Fig. 5 *B* and *C*), suggesting that RHAMM actively suppresses p21 at the posttranscriptional level. Together, these results indicate that RHAMM depletion triggers a compensatory or alternative activation of p21, effectively restoring the G1/S checkpoint. We therefore propose that, in resistant context, RHAMM might suppress p21 expression in a p53-independent manner, thereby allowing polyploid cells to bypass cell cycle arrest and sustain the proliferative capacity required for PCC formation.

Moreover, RHAMM knockdown significantly enhanced the luciferase activity of a reporter construct containing the p21 3′-UTR, while leaving the p21 promoter-driven reporter unaffected (Fig. 5*D*). This indicates that RHAMM likely regulates p21 expression posttranscriptionally—possibly through impairing mRNA stability rather than modulating transcriptional initiation. Consistent with this, RNA degradation assays demonstrated that suppression of RHAMM substantially prolonged the half-life of p21 mRNA (Fig. 5*E*), providing direct evidence that RHAMM promotes p21 transcript decay. Importantly, analysis of human breast cancer transcriptomes (GEO dataset) and our patient dataset (*SI Appendix*, Table S3) further confirmed a significant negative correlation between RHAMM and p21 expression (Fig. 5 *F* and *G*), supporting the relevance of this regulatory axis in human breast tumors. Building on these findings, analysis of TCGA data indicated elevated RHAMM expression in WGD^+^ compared to WGD^−^ breast cancers within TP53-wild-type (TP53^WT^) samples (Fig. 5*H*), supporting an association of RHAMM and WGD/polyploidization. Collectively, these findings indicate RHAMM as a key negative posttranscriptional regulator of p21, enabling cell cycle progression and polyploidization through destabilization of a critical checkpoint mRNA (Fig. 5*I*).

### ER Inhibition–Induced Slug Promotes RHAMM Upregulation.

**Fig. 6.**
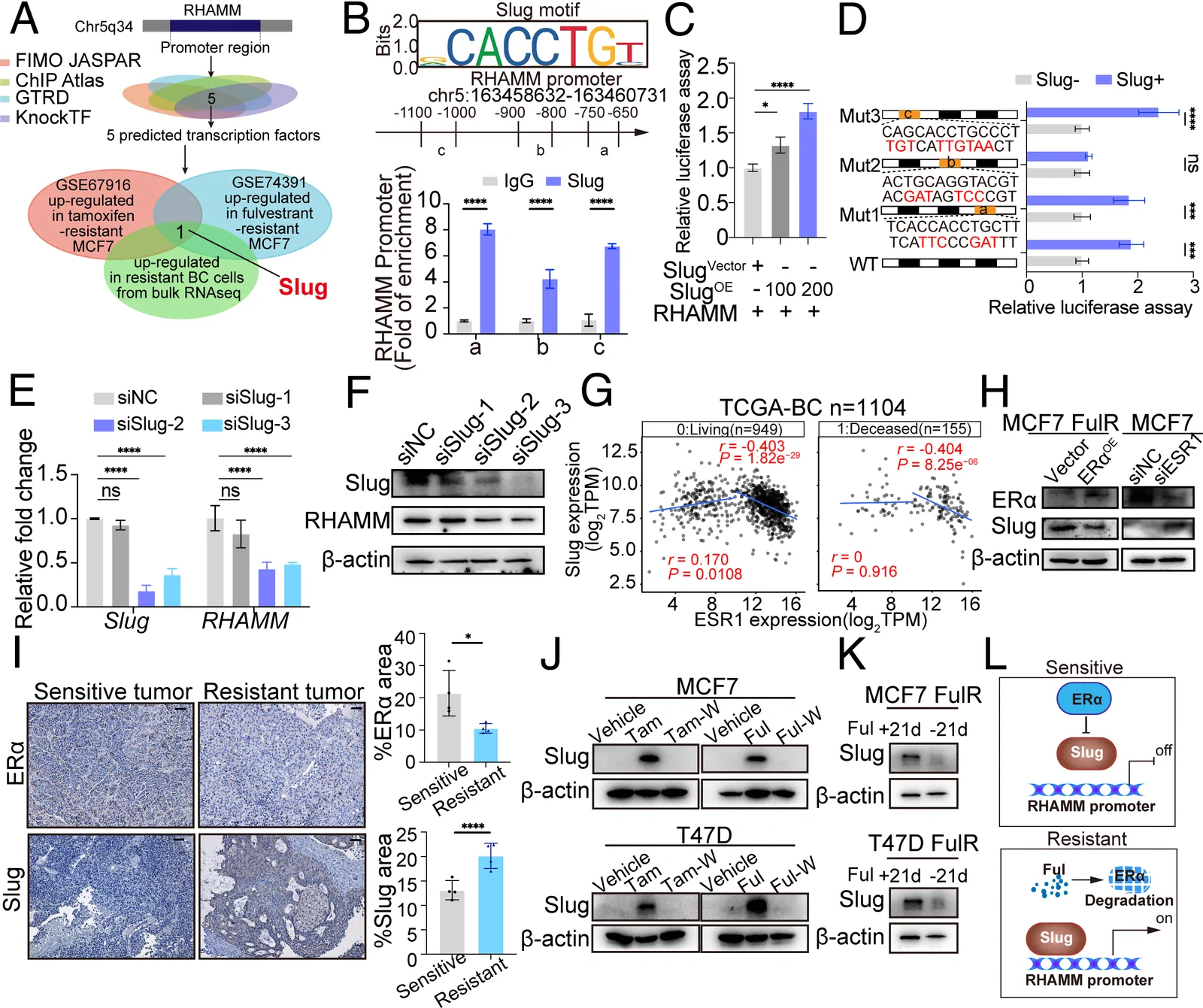
ER inhibition–induced Slug promotes RHAMM upregulation. (*A*) Prediction of RHAMM promoter-binding TFs by public database. The core set of ffive TFs (*JUN, TCF3, SNAI2, NFYB,* and *MITF*) that appeared consistently across all four platforms. Venn diagram of up-regulated DEGs in Tamoxifen (GSE67916) resistant MCF7, fulvestrant resistant MCF7 (GSE74391) and fulvestrant-resistant tumor-derived cancer cells (RNAseq). (*B*) Predicted sequences of Slug binding sites in RHAMM promoter (JASPAR) and their occupancy (ChIP–qPCR) in MCF7/FulR cells across regions 703 to 710, 883 to 891, and 1,055 to 1,067. (*C*) RHAMM promoter activity was measured in 293T cells using a luciferase reporter assay following cotransfection with Slug-overexpression (Slug^OE^) or empty vector (Slug^vector^) plasmids. (*D*) Diagram depicts three JASPAR-predicted Slug binding sites on the RHAMM promoter and their mutated versions used in luciferase reporter assays, and the resulting changes in promoter activity following cotransfection with pCMV/Slug in 293T cells. Three replicate experiments were conducted. (*E* and *F*) Slug’s effect on RHAMM expression in MCF7 FulR cells following siRNA knockdown was assessed using Western blot and RT-qPCR. (*G*) The correlation between Slug and ESR1 expression was observed in 1,104 human breast cancer samples from TCGA. (*H*) Effects of ERα on Slug expression were assessed by immunoblotting in ERα-overexpressing MCF7 cells and ERα-knockdown MCF7 FulR cells. (*I*) IHC staining and quantification of Slug and ERα expression in fulvestrant-sensitive and -resistant tumors (n = 4/group) using the ImageJ IHC Toolbox. (Scale bars, 50 μm.) (*J*) Western blot analysis showed the expression level of Slug in MCF7/T47D cells treated with 1 μM tamoxifen (Tam) or 1 μM fulvestrant (Ful) for 3 d, as well as after 7 d of drug withdrawal (Tam-W, Ful-W). (*K*) Western blot analysis showed the Slug expression levels in the MCF7/T47D fulvestrant-resistant (FulR) cells after 21 d fulvestrant withdrawal. (*L*) Diagram illustrating the mechanism of ERα inhibition-induced Slug mediates RHAMM upregulation. Data represent the mean (±SD). ns (not significant), *P < 0.05, ***P < 0.001, ****P < 0.0001.

To elucidate the molecular mechanisms underlying RHAMM upregulation in fulvestrant-resistant tumors, we predicted transcription factors (TFs) binding to the RHAMM promoter, defined as the region from 2,000 bp upstream to 100 bp downstream of the transcription start site, using JASPAR, GTRD, KnockTF, and ChIP-Atlas. A core set of five TFs consistently identified across all four platforms was then intersected with anti-estrogen-induced up-regulated genes, based on public RNA-seq datasets (GSE67916, GSE74391) and our bulk RNA-seq data from resistant cancer cell (14). This cross-analysis identified SNAI2 (Slug) as a key transcriptional regulator of RHAMM (Fig. 6*A*). Moreover, in large clinical breast cancer cohorts (METABRIC and SCAN-B), patients stratified by median RHAMM and Slug expression levels revealed that the group with high expression of both genes exhibited poorer survival, both in the overall cohort and specifically in ER^+^ breast cancer patients (*SI Appendix*, Fig. S7*A*). Accordingly, immunostaining of primary BC tumors from MMTV-PyMT mice after fulvestrant, letrozole, or tamoxifen treatment for 8 wk revealed that ER suppression by endocrine therapy resulted in a simultaneous increase in RHAMM and Slug levels (*SI Appendix*, Fig. S8). Moreover, RHAMM levels gradually increase with the loss of ESR1 expression from TCGA data analysis based on the molecular subtypes (*SI Appendix*, Fig. S7*B*). Importantly, analysis of our patient dataset further confirmed a significant positive correlation between Slug and RHAMM expression (*SI Appendix*, Fig. S7*C*), supporting the relevance of this regulatory axis in human breast tumors. Therefore, we hypothesized that Slug directly activates RHAMM expression.

To investigate whether Slug directly regulates RHAMM transcription, we first employed JASPAR to predict potential Slug binding sites within the RHAMM promoter, identifying three candidate regions (positions 650 to 750, 800 to 900, and 1,000 to 1,100). Chromatin immunoprecipitation followed by qPCR (ChIP-qPCR) using an anti-Slug antibody in fulvestrant-resistant MCF7 FulR cells confirmed enriched binding of Slug to each of these promoter elements (Fig. 6*B*). To functionally validate the transcriptional effect, we performed luciferase reporter assays in 293T cells and found that Slug overexpression resulted in a dose-dependent increase in RHAMM promoter activity (Fig. 6*C*) and mutation of the Slug binding site at 883 to 891 markedly impaired Slug-induced activation of the RHAMM promoter (Fig. 6*D*), demonstrating that Slug acts as a direct transcriptional activator of RHAMM. Furthermore, knockdown of Slug significantly reduced both mRNA and protein levels of RHAMM in MCF7 FulR cells compared to control cells (Fig. 6 *E* and *F*), providing additional genetic evidence that Slug is necessary for sustained RHAMM expression in the resistant state. Altogether, these results support that the acquirement of a RHAMM^high^ phenotype relies on the Slug pathway.

Slug was up-regulated in fulvestrant-treated breast tumors, prompting us to investigate whether its induction depends on ER status. Analysis of the TCGA breast cancer dataset revealed a context-dependent inverse correlation between SNAI2 (Slug) and ESR1 (Fig. 6*G*). In tumors with high ESR1 expression (log_2_TPM > 10), a significantly negative correlation was consistent with findings in fulvestrant-resistant tumors (Fig. 6*G*). Functional validation showed that ERα overexpression suppressed Slug, whereas ESR1 knockdown enhanced Slug expression (Fig. 6*H*), confirming that ERα negatively regulates Slug. Slug expression was markedly increased in fulvestrant-resistant tumors and in MMTV-PyMT tumors treated with ER inhibitors (fulvestrant, tamoxifen, letrozole), both settings characterized by low ERα levels (Fig. 6*I* and *SI Appendix*, Fig. S8). Notably, Slug levels returned to baseline within 7 d of drug withdrawal in sensitive cells (Fig. 6*J*) and declined after 21 d in resistant models (MCF7 FulR, T47D FulR) (Fig. 6*K*), suggesting this upregulation was reversible. Thus, these findings indicate that the Slug-mediated response to ERα inhibition is a dynamic, reversible adaptive mechanism rather than a permanent genetic switch. Collectively, these findings indicate that ER inhibition activates Slug signaling, which in turn up-regulates RHAMM during acquired endocrine resistance (Fig. 6*L*).

### The RHAMM^high^ Signature Represents a Prognostic Biomarker for Unfavorable Outcomes in ER^+^ BC.

**Fig. 7.**
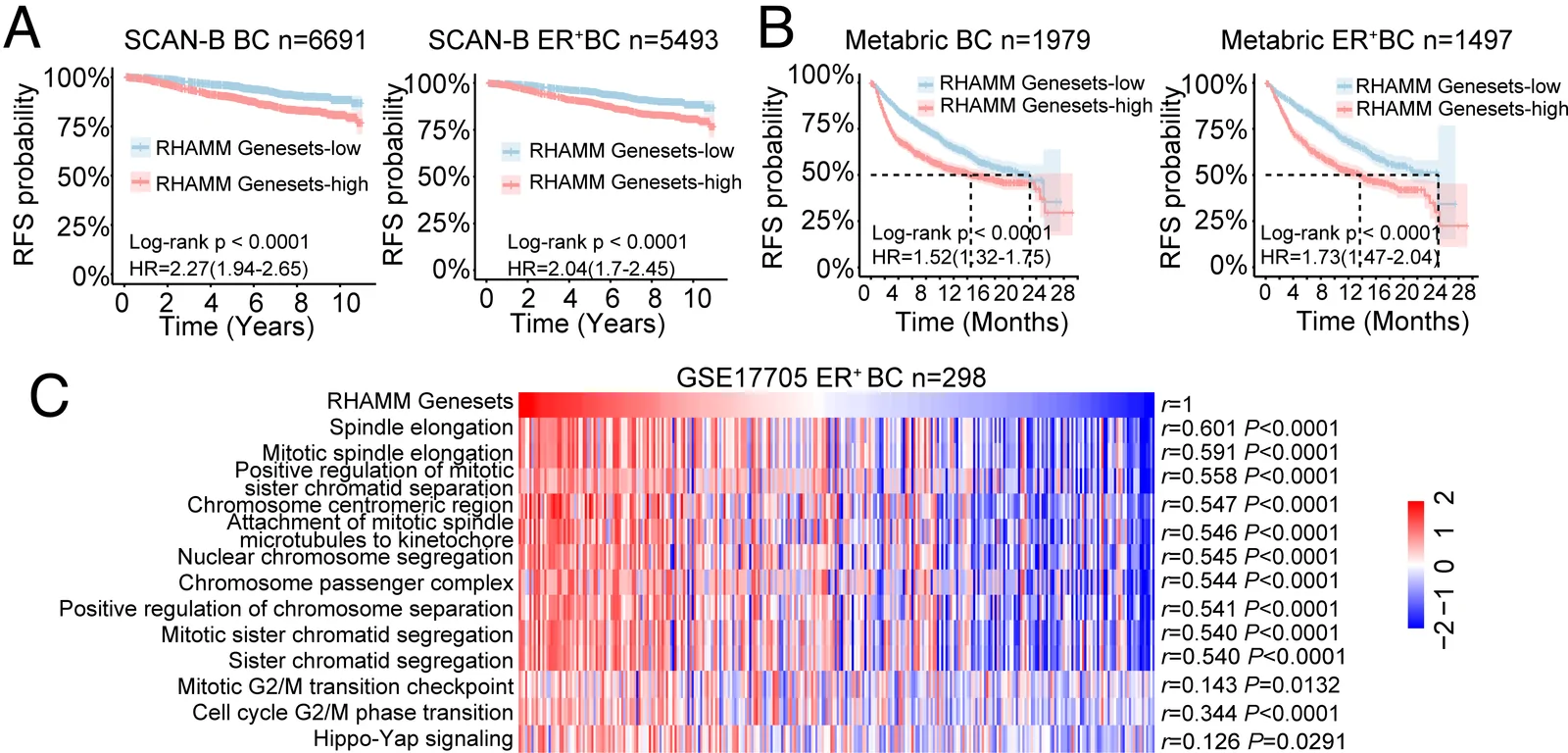
RHAMM^hi^ signature is associated with unfavorable clinical outcomes in ER^+^ BC. (*A* and *B*) KM plot depicted recurrence-free survival (RFS) among all BC and ER^+^ BC patients grouped by the RHAMM gene set signature in SCAN-B database (*A*) and Metabric database (*B*). (*C*) Heatmap depicting correlation signature patterns of the RHAMM gene sets in human ER^+^ BC (GSE17705). Data represent the mean (±SD). ns (not significant), *P < 0.05, ***P < 0.001.

To define the clinical implications of the RHAMM^high^ signature in endocrine resistance, we constructed a 16-gene RHAMM-associated gene set signature by integrating three datasets: highly expressed genes from cluster 6 in scRNA-Seq, RHAMM-interacting proteins identified by co-IP/MS in MCF7 FulR cells and genes down-regulated upon RHAMM knockdown in bulk RNA-seq. In two clinical breast cancer cohorts (METABRIC and SCAN-B), high RHAMM signature scores were significantly associated with poor overall survival in both the total patient population (Fig. 7 *A* and *B*, *Left*) and the ER^+^ breast cancer subgroup (Fig. 7 *A* and *B*, *Right*).

Furthermore, analyses of clinical ER^+^ BC datasets (GSE17705) revealed a positive correlation between the expression of RHAMM-related gene set signature, Hippo-Yap signaling, and various mitosis-related pathways, including spindle elongation, positive regulation of mitotic sister chromatid separation, chromosome centromeric region, the attachment of mitotic spindle microtubules to kinetochore and mitotic G2/M transition checkpoint (Fig. 7*C* and Dataset S5). This finding indicated a strong link between the RHAMM signature, Hippo-YAP signaling, and mitotic regulation.

Together, these multilevel analyses strongly implicate RHAMM as a key mediator of tumor aggression and therapy resistance in ER^+^ breast cancer, and highlight its potential as a prognostic biomarker and therapeutic target.

### Repression of RHAMM Inhibits the Formation of PCCs and Improves Fulvestrant Efficiency.

To investigate whether RHAMM plays a direct functional role in endocrine resistance, we first knocked down RHAMM in fulvestrant-resistant cells (*SI Appendix*, Fig. S9*A*). This resulted in a marked increase in drug sensitivity relative to controls (*SI Appendix*, Fig. S9*B*), along with reduced expression of c-myc and Ki67 (*SI Appendix*, Fig. S9 *C* and *D*), suggesting a potential link between RHAMM and cell proliferation.

**Fig. 8.**
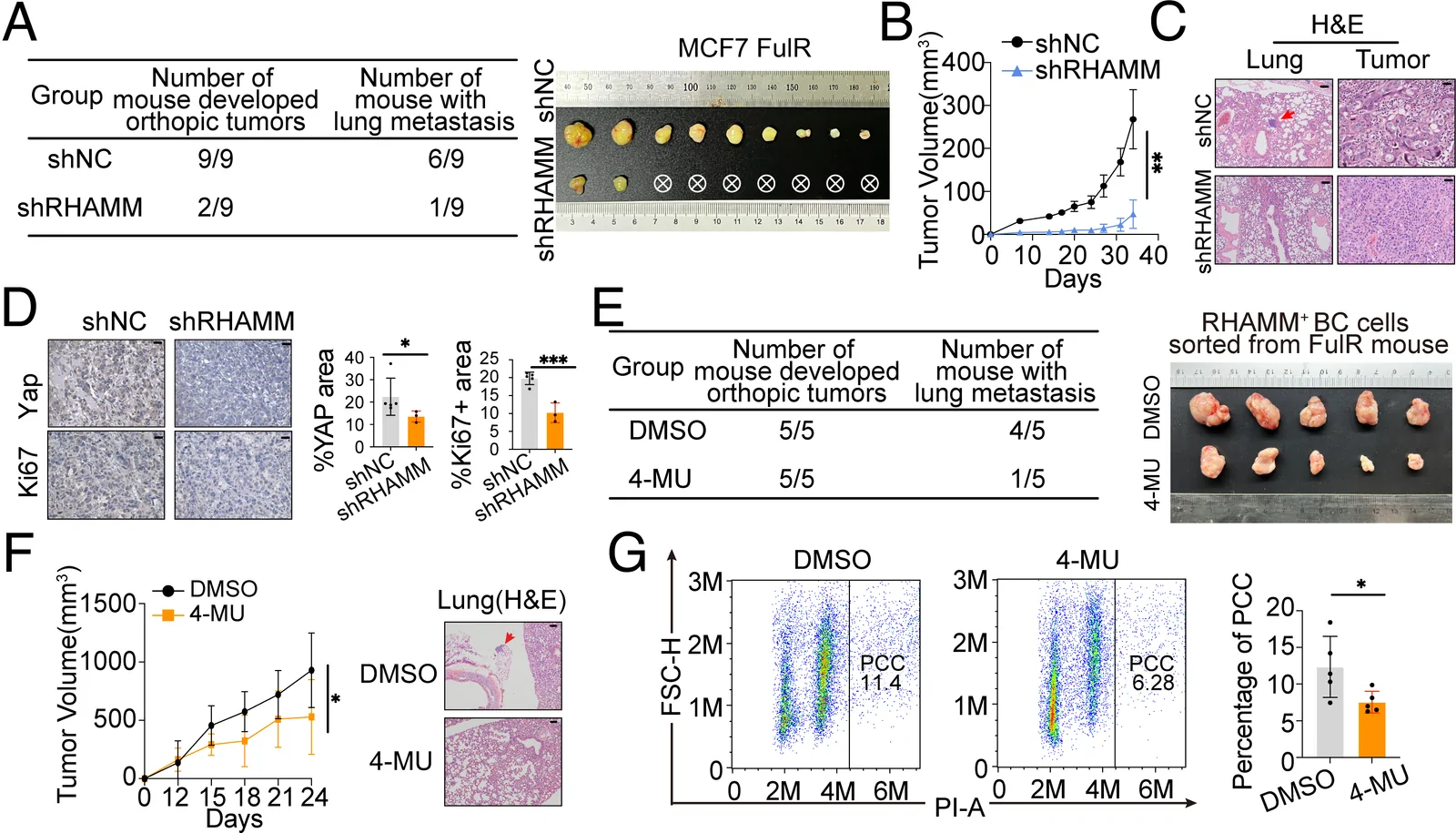
Blocking PCCs development improves endocrine therapy efficiency. (*A*) The development of orthotopic xenografts using resistant BC cells (MCF7 FulR^shNC^ vs. MCF7 FulR^shRHAMM^) upon treatment with fulvestrant (50 mg/kg, i.m.). Images of the developed orthotopic xenografts and the number of mice that developed orthotopic tumors and metastases. (*B*) Tumor volume of orthotopic xenografts in (*A*). (*C*) Representative HE staining of lung and tumor tissue. (Scale bar, *Top*: 50 μm, *Bottom*: 20 μm.) (*D*) IHC analysis of YAP and Ki67 expression in xenografts from (*A*). Positive staining was evaluated using the ImageJ IHC Toolbox plugin. (Scale bars, 50 μm.) (*E*) RHAMM^+^ tumor cells from fulvestrant-resistant breast tumors were orthotopically implanted into Fvb mice. Once tumors reached a volume of 100 mm^3^, mice received 4MU [400 mg/kg, oral administration (p.o.)] and fulvestant (50 mg/kg, i.m.) treatment. Xenograft images and counts of mice with tumors and metastases. (*F*) *Left*, Tumor growth of orthotopic xenografts in (*E*). *Right*, representative H&E staining of lung sections. (Scale bar, 50 μm.) (*G*) Cells isolated from tumors in (*E*) were subjected to polyploidy analysis using PI DNA staining. Data represent the mean (±SD). *P < 0.05, **P < 0.01, ***P < 0.001.

We next asked whether RHAMM depletion could reverse therapy resistance in vivo. To address this, RHAMM^+^ cells isolated from MCF7 FulR cells via FACS were transduced with lentiviral shRNA targeting RHAMM and orthotopically implanted into immunodeficient mice under fulvestrant treatment. RHAMM knockdown not only attenuated tumor growth and restored fulvestrant sensitivity (Fig. 8 *A* and *B*) but also significantly suppressed the formation of lung metastatic foci and the expression of Yap and Ki67 (Fig. 8 *C* and *D*).

Given that 4-MU down-regulates RHAMM expression, we further evaluated its therapeutic potential in a syngeneic setting. RHAMM^+^ cells isolated from resistant murine tumors were transplanted into wild-type Fvb mice, which subsequently received combination therapy with 4-MU and fulvestrant. 4-MU treatment significantly inhibited tumor proliferation and distant metastasis compared to control groups (Fig. 8 *E* and *F*). Consistent with these phenotypic outcomes, PI staining revealed a pronounced reduction in the PCC population in 4-MU-treated tumors (Fig. 8*G*), supporting a mechanism wherein RHAMM fosters therapy resistance and metastasis—potentially by facilitating polyploidization and cellular survival.

These findings indicate that RHAMM deletion suppresses PCC formation, impairs tumorigenesis in resistant breast cancer, thereby enhancing the efficacy of fulvestrant. Collectively, our study identifies RHAMM as a key mediator of acquired antiestrogen resistance in breast cancer and suggests that cotargeting RHAMM in combination with fulvestrant may represent a promising therapeutic strategy.

## Discussion

The mechanisms through which diploid tumors evolve into polyploid cell populations remain poorly understood. In this study, we identify RHAMM as a critical driver of endocrine resistance in ER^+^ breast cancer by facilitating the formation of PCCs, thereby uncovering a previously uncharacterized mechanism underlying polyploidization. Single-cell transcriptomic analysis identified RHAMM as a hallmark feature of a distinct G2/M-enriched subpopulation in fulvestrant-resistant tumors, where it functionally orchestrates the development of PCCs—a phenotype strongly associated with therapy resistance and adaptive plasticity in multiple cancer types. Mechanistically, ER inhibition triggers Slug activation, which up-regulates RHAMM expression. RHAMM in turn facilitates the recruitment of Septin complexes, promotes microtubule assembly, and induces cytoskeletal reorganization, collectively leading to YAP activation and the induction of polyploidy. Together, RHAMM upregulation promotes genomic instability through two synergistic mechanisms: inducing cytokinesis failure via aberrant cytoskeletal polymerization and impairing the G1/S checkpoint through accelerated p21 degradation. These processes collectively facilitate polyploidization and drive tumorigenesis. Importantly, RHAMM^high^ signatures were significantly enriched in metastatic and recurrent ER^+^ breast tumors with aggressive clinicopathological features and poor outcomes, underscoring its translational relevance as a potential therapeutic target, as corroborated by Tarullo et al., who linked elevated RHAMM to an invasive niche and poor prognosis (11). Although PCCs have previously been implicated in chemotherapy resistance, our work provides causal evidence linking RHAMM-driven polyploidization as a specific adaptive strategy contributing to endocrine treatment failure in ER^+^ breast cancer.

Although PCCs were once overlooked or even mistaken for “dead cells” due to their inability to undergo conventional mitosis, emerging evidence indicates that they can generate therapy-resistant progeny through nuclear budding or bursting (30, 31). Our findings expand this paradigm by revealing a dynamic, iterative evolution of PCCs. After an initial cytokinesis failure, these cells often reenter cell cycle, but subsequent divisions are frequently aberrant, producing “ring-shaped” (wreath-like) nuclei (Movie S3). Moreover, a single large PCC can undergo asymmetric cell division, yielding one large daughter PCC and several smaller, viable progeny cells (Movie S4), suggesting that PCCs act as specialized reproductive hubs rather than static entities. PCCs have been implicated in resistance across multiple cancers (32–36). In our endocrine-resistant ER^+^ breast cancer model, PCCs constitute ≈10% of the cell population—a frequency similar to that reported in colorectal radiation-tolerant persisters (≈10%) (37) and olaparib-resistant breast cancer cells (9.77%) (38), but lower than in olaparib-resistant ovarian cancer (16.9 to 34.4%) (38). This disparity likely reflects differences in treatment and tumor context, emphasizing the need for cancer-specific evaluation of PCC contributions to resistance.

While the involvement of RHAMM in cytoskeletal regulation has been previously documented—notably through ROCK-mediated stabilization of F-actin polymerization in prostate cancer (39)—our study uncovers a mechanistic pathway. We demonstrate that RHAMM directly interacts with Septin9/10, leading to enhanced actin polymerization and microtubule stabilization, which collectively drive profound cytoskeletal dysregulation. This abnormal cytoskeleton polymerization facilitates YAP activation and subsequent cytokinesis failure. Notably, RHAMM’s engagement with septins extends its functional scope beyond canonical hyaluronan-dependent signaling pathways. The resulting RHAMM–YAP axis creates a permissive microenvironment for the formation of PCCs, as supported by a marked reduction in PCC generation following either YAP inhibition (e.g., with verteporfin) or RHAMM suppression (e.g., via 4-MU). Our findings implicate YAP as a critical regulator of polyploid cell survival, aligning with its documented role in polyploidization. Previous studies indicate that YAP expression is elevated in tetraploid cells, while the Hippo kinase Last2 counteracts YAP activity by stabilizing p53, thereby exerting tumor-suppressive effects (40). Furthermore, YAP–P27-mediated ploidy regulation has been shown to mitigate genomic instability and promote tumorigenesis in hepatic contexts (41). Our study and these previous observations underscore a context-dependent interplay between YAP activation and polyploidization, highlighting both oncogenic and potentially adaptive functions within stressed tumor environments.

Following cytokinesis failure induced by RHAMM, p53-proficient resistant cells did not undergo DNA damage related cell cycle arrest. This observation aligns with prior studies demonstrating that Cyclin E overexpression bypasses senescence and promotes endoreduplication (18). Consistent with these findings, our data indicate that PCCs with impaired p21 signaling are predisposed to bypass cell cycle arrest and undergo reduplication. We propose that RHAMM acts as a key posttranscriptional regulator of p21 mRNA. Given the well-established role of p21 in enforcing senescence at cell cycle checkpoints, we speculate that RHAMM-mediated suppression of p21 may facilitate checkpoint bypass, allowing PCCs to escape cell cycle arrest and survive. In RHAMMhigh resistant cells, cytokinesis failure may initially trigger arrest, but subsequent dysregulation of the p21 pathway could permit escape from arrest and reentry cell cycle.

While previous studies indicate that polyploidization/WGD depends on p53 deficiency or p53 activation under cyclin E-mediated replicative stress (18), we find that acquired RHAMM upregulation drives polyploidization in endocrine-resistant BC cells independently of p53—though p53 is highly expressed. Thus, we speculate that this discrepancy can be attributed to distinct underlying mechanisms: the double-strand breaks (DSB)-induced WGD requires p53 loss to bypass DNA damage-induced G1 arrest, as p53-mediated G1 arrest suppresses WGD. In the context of cyclin E-induced replicative stress (18), WGD relies on p53 proficiency to enable p21-mediated CDK inhibition and mitotic bypass. However, the mechanism of RHAMM-driven polyploidization we propose operates regardless of p53 status, as our results illustrate loss of p21-decoupled from p53 regulation-is sufficient to abrogate G1 arrest and promotes polyploidization in fulvestrant-resistant BC cells. Since polyploidization itself fails to trigger robust p53-dependent arrest, p53 deficiency becomes unnecessary (5). Thus, RHAMM-mediated polyploidization circumvents canonical p53 constraints through p21 inactivation, highlighting a route to genome duplication in fulvestrant-resistant BC cells.

Previous studies have linked elevated Slug expression to an aggressive phenotype in fulvestrant-resistant breast cancer cells and poor endocrine therapy response in metastatic ER^+^ BC (42). In this study, starting with the observation of high RHAMM expression, we identified Slug as the upstream molecule regulating RHAMM transcription. Furthermore, existing studies indicate that ER negatively correlates with Slug and directly suppresses its transcription to modulate EMT (43). Consistent with these reports, our findings also show a negative correlation between ER and Slug levels. Here, we demonstrate that ER inhibition up-regulates Slug, which in turn directly activates RHAMM transcription, thereby revealing a previously undescribed ER–Slug–RHAMM regulatory axis. This pathway illustrates how endocrine therapy resistance is mediated through Slug-driven transcriptional reprogramming. Ultimately, RHAMM upregulation enhances cellular plasticity, enabling resistance to antiestrogen treatment through polyploidization and polyploid cell formation.

In summary, we propose that the targeting of RHAMM–YAP axis or PCC formation in combination with endocrine treatments has therapeutic potential and is likely to improve treatment outcomes for advanced BC patients. It is important to emphasize, however, that these findings remain preliminary; further rigorous experimentation is necessary to validate the proposed mechanism. The clinical relevance of the polyploidization mechanism we described in this study in acquired endocrine resistance—particularly the gradual upregulation of RHAMM during therapy—needs to be validated in human studies.

## Materials and Methods

### Human Tumor Tissues and Tissue Microarray Analysis.

Human tumor tissues were analyzed using a tissue microarray (HBreD140Su04, 134 human breast lesions, Shanghai Superchip Biotech) to assess RHAMM expression frequency across molecular subtypes, with clinicopathological characteristics acquired from medical records (*SI Appendix*, Table S2); additionally, RHAMM, P21, and Slug expression was examined in a small cohort of human luminal-like breast tumors from patients with or without lymph node metastasis (non-LNM n = 9, LNM n = 9, *SI Appendix*, Table S3). The study protocol involving human tissue samples was approved by the ethical committee of Shanghai Jiao Tong University Affiliated Sixth People’s Hospital (2021-183). Informed consent was obtained from all participants.

Please refer to *SI Appendix*, *Supplementary Materials and Methods* for the detailed methods on antibodies and reagents, mice, time-lapse cell cycle imaging, WGD status, tumor purity, and ploidy for TCGA samples, microarray data analysis, statistical analysis, cell culture, single-cell data integration and analysis, IHC, colony formation, FRAP, image acquisition and quantification, flow cytometry, ChIP-qPCR, siRNA and shRNA transfection, co-IP/MS, cell proliferation, immunofluorescence, RNA sequencing, western blot, RT-PCR, dual-luciferase reporter assay, and spheroid formation.

## Ethical Approval

All animal experiment protocols were approved by Institutional Animal Care and Use Committee and all procedures were performed in compliance with relevant laws and institutional guidelines.

## Supplementary Material

Appendix 01 (PDF)

Dataset S01 (XLSX)

Dataset S02 (XLSX)

Dataset S03 (XLSX)

Dataset S04 (XLSX)

Dataset S05 (XLSX)

Movie S1. **Formation of a PCC via cytokinesis failure in MCF7 FulR^shNC^ cells**. Time-lapse imaging shows a representative sequence of PCC formation in control (shNC) cells. A progenitor cell attempts mitosis but undergoes cytokinesis failure, resulting in the development of a large, multinucleated/polyploid cell. This video corresponds to the representative stages shown in Fig. 3D (top). Images were captured every 30 minutes using the FUCCI system to monitor cell cycle progression.

Movie S2. **Conventional bipolar mitosis in RHAMM-knockdown MCF7 FulR cells**. Time-lapse imaging demonstrates the typical cell division pattern in RHAMM-deficient (shRHAMM) cells. Unlike the control group, these cells predominantly undergo successful bipolar mitosis, resulting in two distinct daughter cells. This video corresponds to the representative sequence shown in Fig. 3D (bottom).

Movie S3. **Development and subsequent division of a rosette-like PCC in MCF7 FulR^shNC^cells**. This video highlights the dynamic formation of a PCC through an alternative route. Following a bipolar division, a daughter cell exhibits an abnormal cell cycle, leading to the formation of a PCC with a characteristic “rosette-like” nuclear morphology. Notably, this newly formed PCC is shown undergoing a subsequent round of cell division.

Movie S4. **Complex division of a PCC into multiple progeny in MCF7 FulR^shNC^ cells**. Time-lapse imaging illustrates a representative “reductive” or asymmetric division of a PCC. The large mother cell undergoes a multi-polar or irregular fission process, generating a new PCC alongside numerous smaller, viable-appearing progeny cells.

Movie S5. **Abnormal multipolar division of a PCC in MCF7 FulR^shNC^ cells**. Time-lapse fluorescence imaging (related to Fig. 3H, left) reveals the aberrant mitotic machinery in a representative PCC from the shNC group. The video tracks the dynamics of Centromere B (Cenpb-EGFP, green) and α-Tubulin (mCherry, red). The PCC exhibits highly disorganized spindle assembly and irregular chromosome segregation, leading to a complex, non-canonical mitotic event. Images were captured every 3 minutes to provide high-temporal resolution of the mitotic failure and subsequent fragmentation.

Movie S6. **Classical bipolar mitosis in RHAMM-knockdown MCF7 FulR cells**. Time-lapse fluorescence imaging (related to Fig. 3H, right) shows the standardized cell division process in shRHAMM cells expressing Cenpb-EGFP (green) and Tubulin-mCherry (red). RHAMM-knockdown cells demonstrate precise bipolar spindle formation and symmetrical alignment and segregation of centromeres, resulting in a successful classical mitotic division.

## Data Availability

The raw RNA sequence data have been deposited in the Genome Sequence Archive at the National Genomics Data Center (HRA020423) (44). The Co-IP-based mass spectrometry proteomics data have been deposited in the OMIX database (OMIX019775) (45). All other data are included in the manuscript and/or supporting information. Previously published data were used for this work (14).
