# Supplementary material for: RHAMM drives formation of polyploid cancer cells and confers resistance to ER-targeted therapy in breast cancer
Source: Proc Natl Acad Sci U S A. 2026 Sep 16;123(38):e2535342123. doi: 10.1073/pnas.2535342123 (PMC13598241; doi:10.1073/pnas.2535342123)
Supplement: Supplementary file 1 [file pnas.2535342123.sapp.pdf]

SUPPLEMENTARY INFORMATION

– Supplemental Figures and Tables

– Supplementary Movie Legends

– Supplementary Materials and Methods

Supplemental Figures and Tables

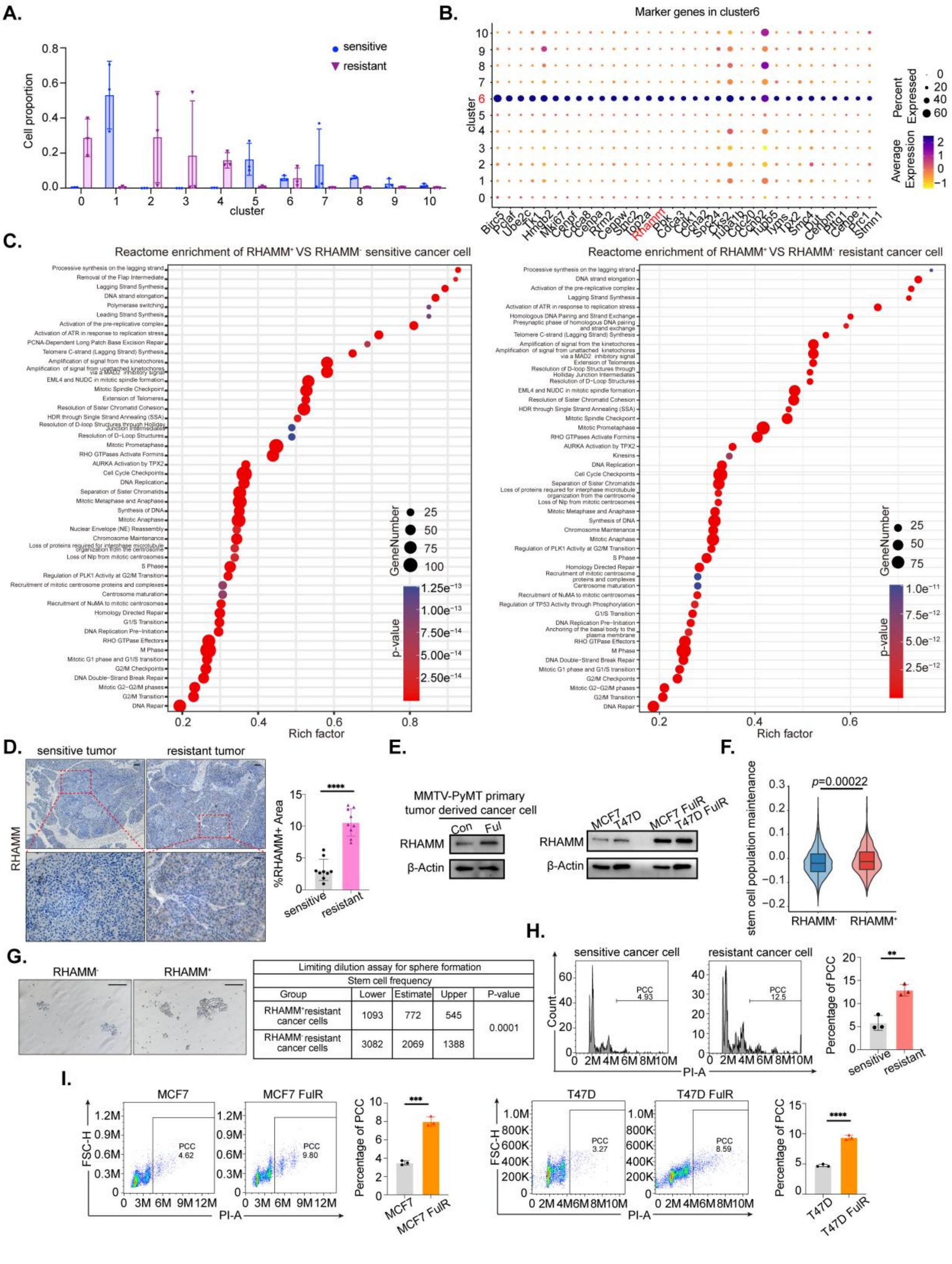

Fig.S1 RHAMM+ cancer cells in the developing endocrine resistant BCs

(A) Bar plot showing the proportions of clusters in sensitive and resistant group. **(B)**Dotplot showing the relative expression of the most enriched genes for cluster 6. **(C)**Reactome enrichment heatmap comparing RHAMM$^+$ and RHAMM$^-$ cancer cells in both sensitive and resistant group.**(D)**RHAMM expression was assessed by immunohistochemistry (IHC) in fulvestrant-sensitive and -resistant tumors (n=9 per group) and quantified using the ImageJ IHC Toolbox. Scale bar, top: 100μm, bottom: 20μm.**(E)**Western blotting was performed to analyze RHAMM expression in cancer cells from fulvestrant-treated mouse BC models, their parental counterparts, and fulvestrant-resistant cell lines.**(F)**Violin plot depicting geneset score of stem cell population maintenance comparing RHAMM$^+$ and RHAMM$^-$ resistant cancer cells from scRNA-seq. **(G)**Mammosphere-forming capacity of RHAMM$^{-/+}$ fulvestrant-resistant BC cells. The frequency of tumor-initiating cells in the RHAMM$^{-/+}$ fulvestrant-resistant BC cells was calculated using the Extreme Limiting Dilution Analysis Program. Scale bars: 100μm.**(H)**PI staining-based analysis of changes in the proportion of PCCs in primary fulvestrant-sensitive and -resistant cancer cells.**(I)**PI staining-based analysis of changes in the proportion of PCCs in MCF7/T47D FulR cells. Data are presented as mean (±SD) from three independent experiments. $^{**}P < 0.01$, $^{****}P < 0.0001$.

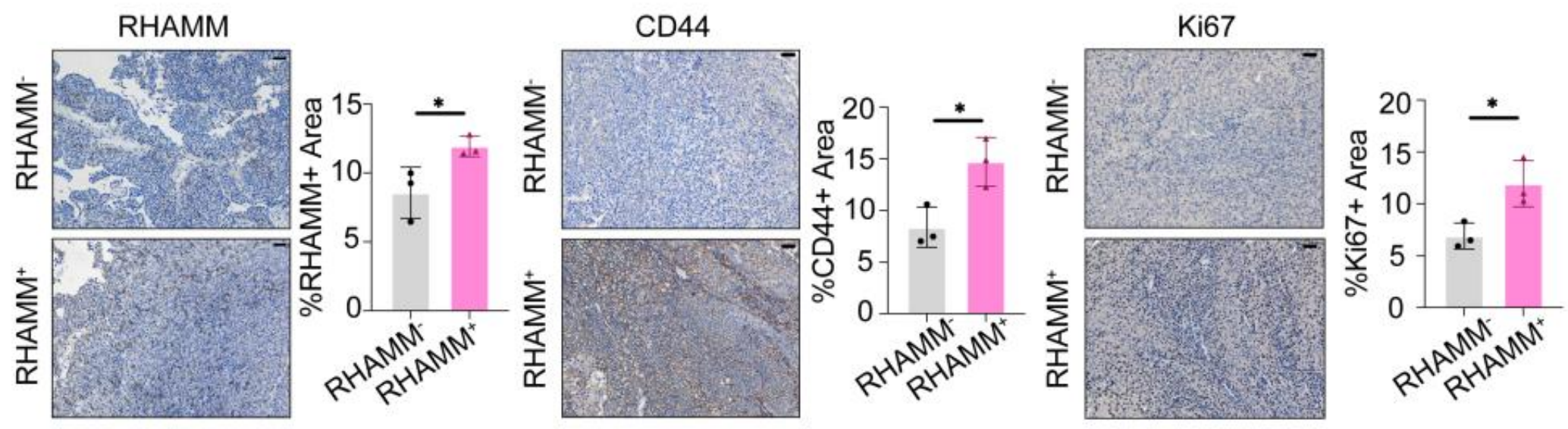

Fig. S2 RHAMM$^+$ cancer cells exhibit increased resistance to endocrine therapy

IHC was performed to assess RHAMM, CD44, and Ki67 expression in transplanted tumors derived from RHAMM$^{-/+}$ fulvestrant-resistant cancer cells. The positive staining indices were quantified using the ImageJ IHC Toolbox software. Scale bars: 50μm. Data represent the mean (±SD). $^{*}P < 0.05$.

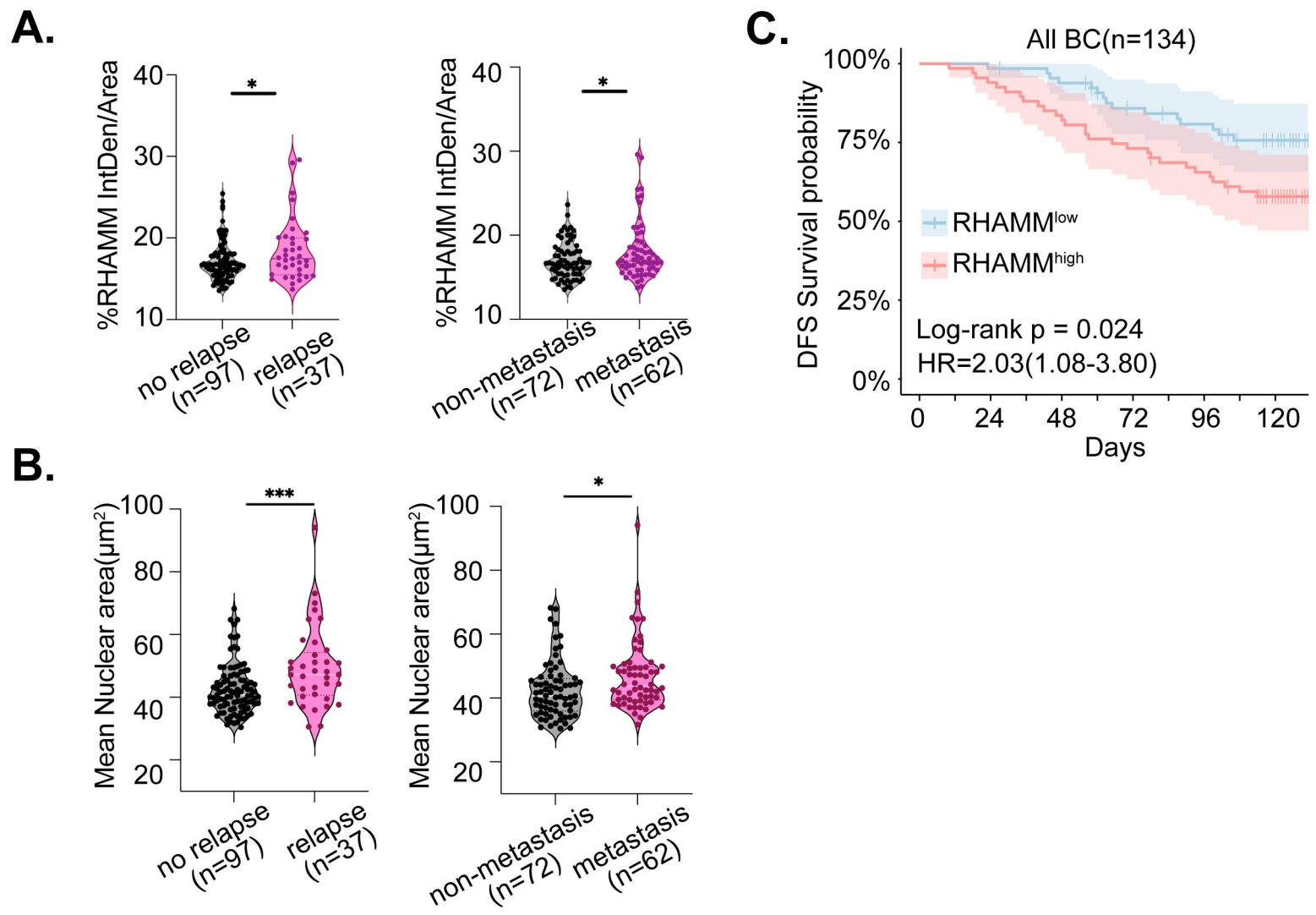

Fig. S3 High RHAMM expression correlates with poor clinical outcomes in cancer patients.

(A & B) Violin plot depicting RHAMM expression (A) and quantification of nuclear area (B) in primary BC tissues(n=134) stratified by relapse (no relapse, n = 97; relapse, n = 37) and metastasis (non-metastasis, n=72; metastasis, n=62). **(C)**Survival probability analysis (Disease-free survival, DFS) of all breast cancer patients (n=134) stratified by high (n = 67) vs. low (n = 67) RHAMM expression. BC: breast cancer. **P* < 0.05 , ****P* < 0.001.

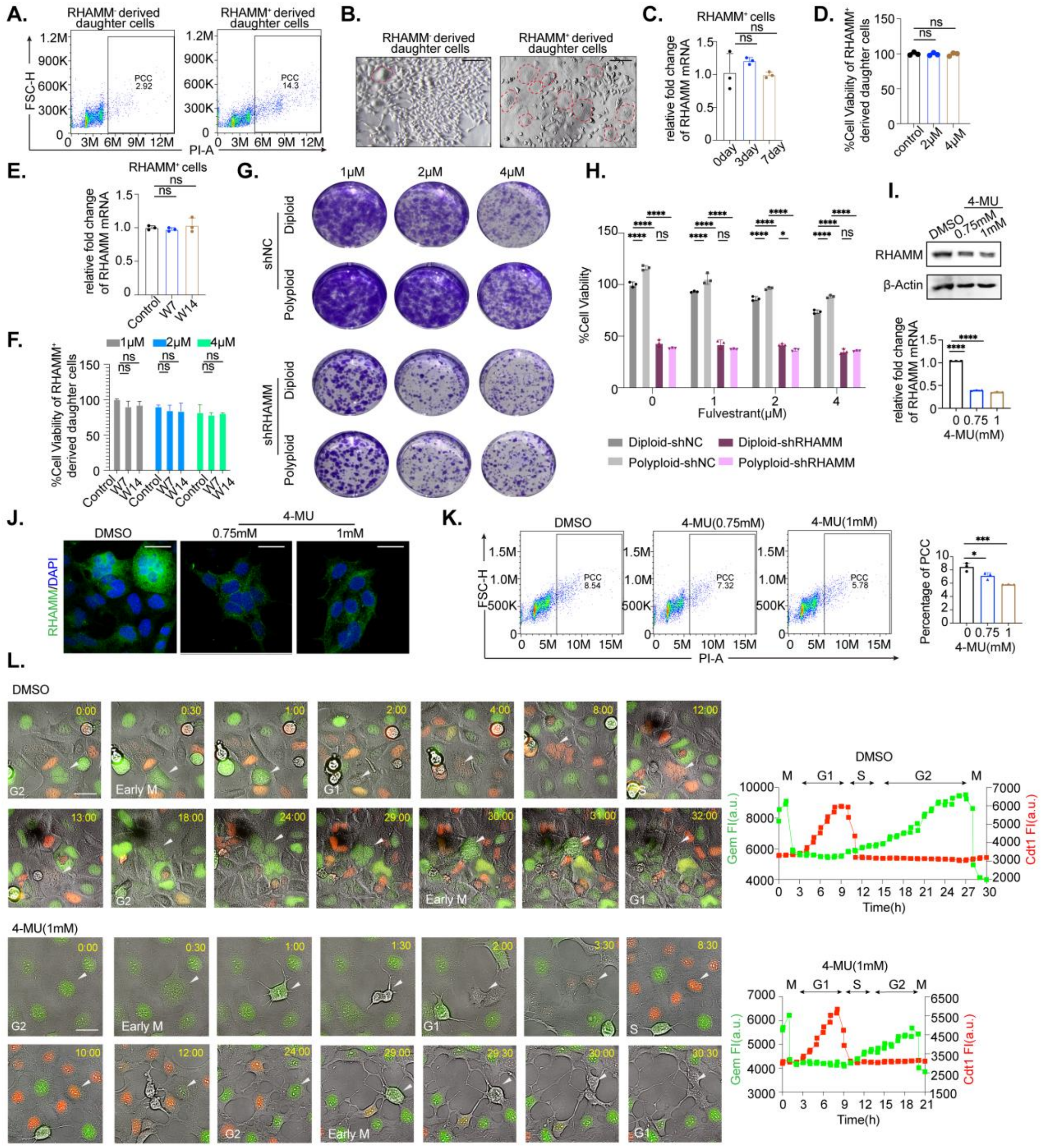

Fig. S4 RHAMM contributes to the accumulation of PCCs in fulvestrant-resistant BC

(A)PI staining-based analysis of proportion of PCCs in daughter cells of RHAMM$^{-/+}$ single clone sorted from MCF7 FulR . **(B)**Representative phase-contrast microscopy images of daughter cells of RHAMM$^{-/+}$ single clone sorted from MCF7 FulR. Scale bars, 100μm.**(C)**RT-qPCR analysis of RHAMM expression in RHAMM^{+} cell clone sorted from MCF7 FulR after culturing.**(D)**CCK8 assays of cell viability under fulvestrant treatment for 72 hours. **(E)** RT-PCR analysis of RHAMM mRNA levels in daughter cells of RHAMM^{+} cells at 7 and 14 days after fulvestrant withdrawal. **(F)**Daughter cells of RHAMM^{+} cells were cultured in fulvestrant-free medium for 7 or 14 days, followed by a 3-day treatment with fulvestrant at the indicated doses. Cell viability was assessed using CCK-8 assays. **(G)**Colony formation assays performed on diploid and polyploid cancer cell (PCC) populations isolated from MCF7 FulR shNC and shRHAMM cells in the presence of fulvestrant. **(H)**Cell viability measured by CCK-8 assay in diploid and polyploid MCF7 FulR cells

after 3 days of fulvestrant exposure. **(I)**Western blot and RT-qPCR and **(J)**Immunofluorescence staining analysis of RHAMM expression in MCF7 FulR cells after treated with 0.75mM or 1mM 4-MU (4-methylumbelliferone) for 72 hours. Scale bars, 50μm. **(K)**PI staining-based analysis of changes in the proportion of PCCs in MCF7 FulR cells after treated with 4-MU for 72 hours. Data are presented as mean (±SD) from three independent experiments. **(L)**Tracking of cell cycle changes in MCF7 FulR cells exposed to 0.75mM 4-MU for 72 hours. Scale bars, 50μm. Temporal profiles of fluorescence intensities (FI) of mCherry-cdt1 and mVenus-Gem of cells. a.u., arbitrary unit. Data represent the mean (±SD). *$P < 0.05$, ***$P < 0.001$, ****$P < 0.0001$.

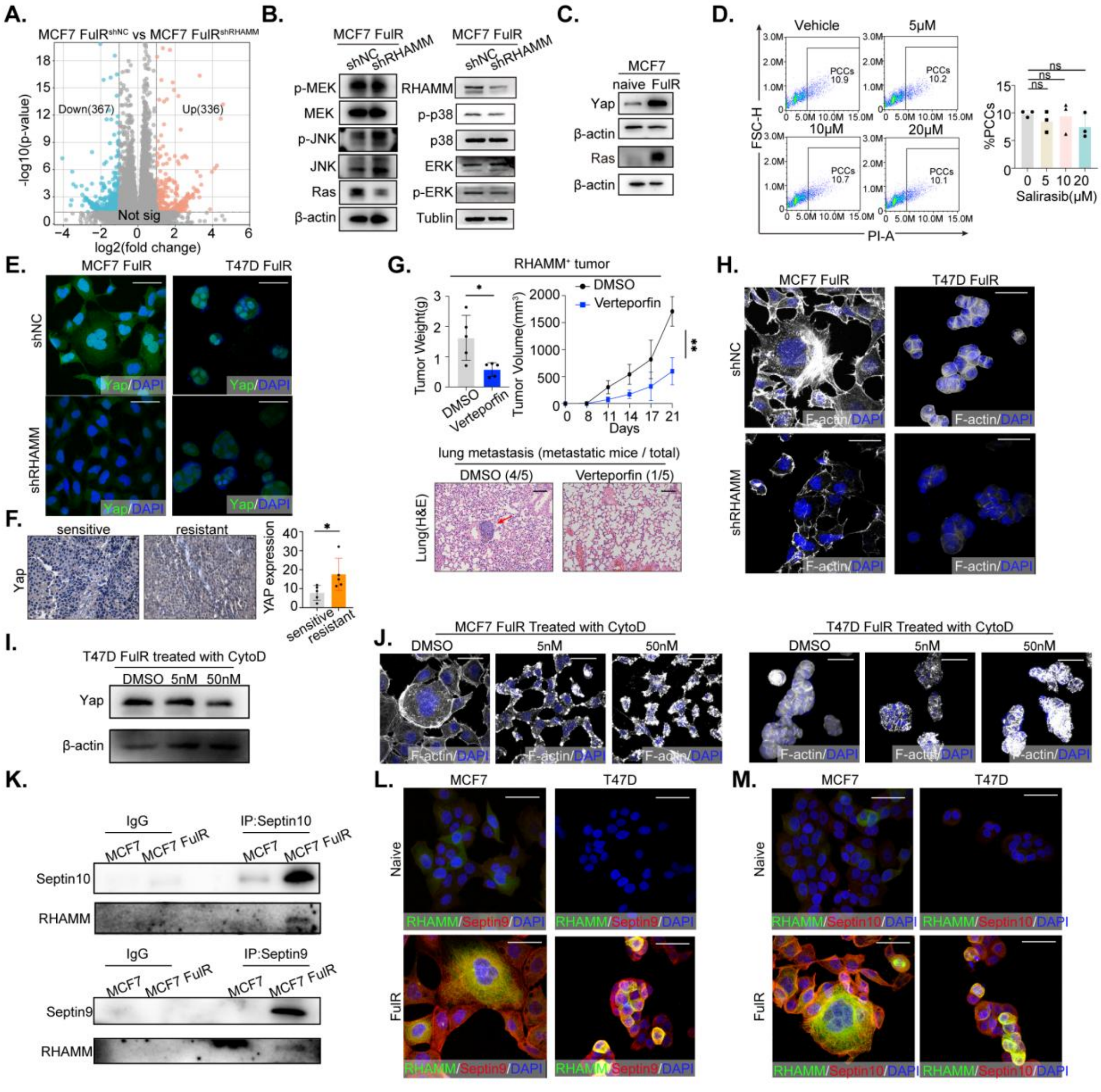

Fig. S5 RHAMM activates YAP signaling to convert cancer cells into PCCs

(A) Volcano plots showing differentially expressed genes between MCF7 FulR^{shNC} and MCF7 FulR^{shRHAMM} cells (RNA-seq data; |log2FC| > 1 and FDR < 0.01). **(B)**Western blot analysis of the MAPK pathway in MCF7 FulR^{shNC} and MCF7 FulR^{shRHAMM} cells. **(C)**Western blot analysis of YAP and Ras expression in MCF7/MCF7 FulR cells. **(D)**PI staining-based analysis of the PCCs proportion in MCF7 FulR cells treated with Salirasib for 48 hours. Data are presented as mean (±SD) from three independent experiments. **(E)**Immunofluorescence staining showing alterations in YAP expression in MCF7/T47D FulR cells after RHAMM knockdown. Scale bars: 50 μm.

(F)IHC analysis of YAP expression in fulvestrant-sensitive and -resistant BCs. YAP staining positivity was evaluated using the ImageJ IHC Toolbox plugin. Scale bars, 20μm. **(G)**Top, tumor weight and tumor growth of orthotopic transplants from resistant-RHAMM^{+} cancer cells (related to Fig.4D). Bottom, representative H&E staining of lung sections from Figure 3D. Scale bars, 50μm. **(H)**RHAMM knockdown effect on F-actin expression and distribution in fulvestrant-resistant BC cells (MCF7/T47D FulR). Scale bars: 50 μm. **(I)**Western blot analysis of YAP expression in T47D FulR after treated with cytochalasin D(CytoD; 5nM, 50nM) for 24 hours. **(J)**Immunofluorescence staining of F-actin expression and distribution in fulvestrant-resistant BC cells after 24-hour CytoD treatment. Scale bars, 50μm. **(K)**Co-immunoprecipitation of RHAMM and septin9/10 interactions in MCF7 and MCF7 FulR cells. **(L&M)**Immunofluorescence staining was used to analyze the co-localization of RHAMM(green) and septin9/10(red) in fulvestrant-resistant and parental MCF7/T47D cells. Scale bars, 50μm. Data represent the mean (±SD). ns (not significant), *P < 0.05 , **P< 0.01.

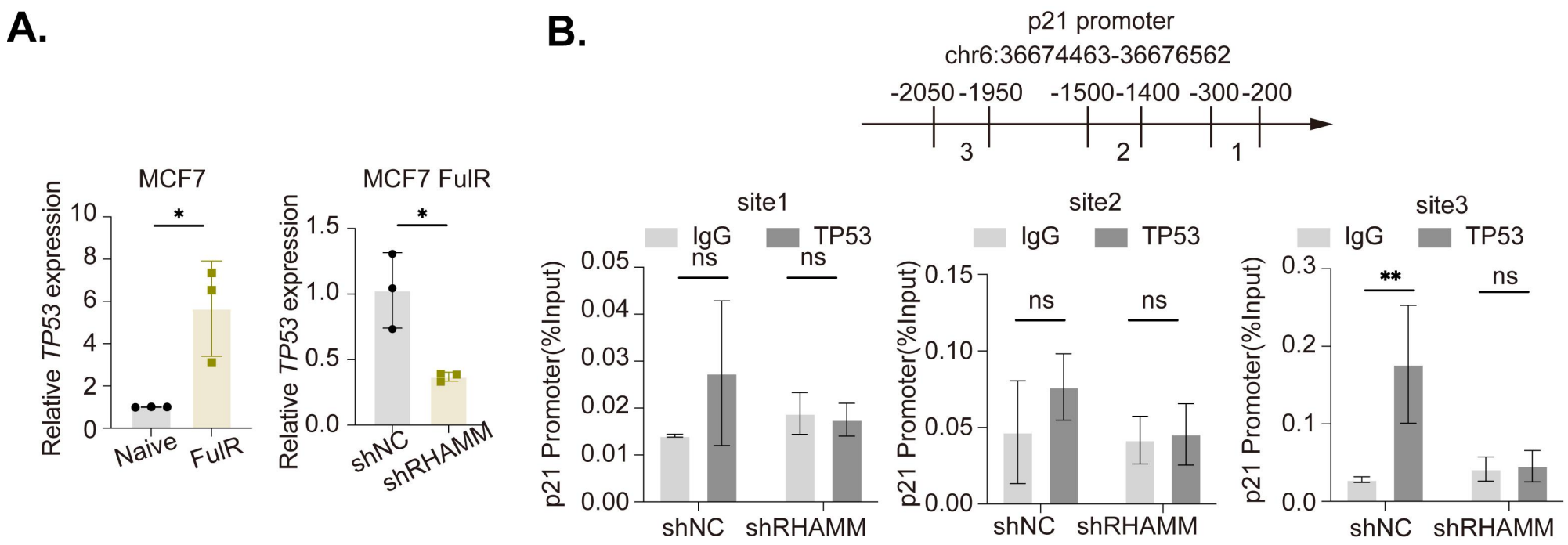

Fig. S6 Validation of p53 expression and its transcriptional activity at the p21 promoter.

(A)RT-qPCR analysis of TP53 mRNA expression levels in MCF7 naïve, MCF7 FulR, MCF7 FulR shNC, and MCF7 FulR shRHAMM cells. **(B)**Predicted TP53 binding sites in the p21 promoter (JASPAR) and their occupancy at regions 279-296, 1419-1436, and 1990-2004. ChIP-qPCR analysis illustrating p53 occupancy at these regions before and after RHAMM knockdown in MCF7 FulR cells. ns: no significance, *P < 0.05 , **P < 0.01.

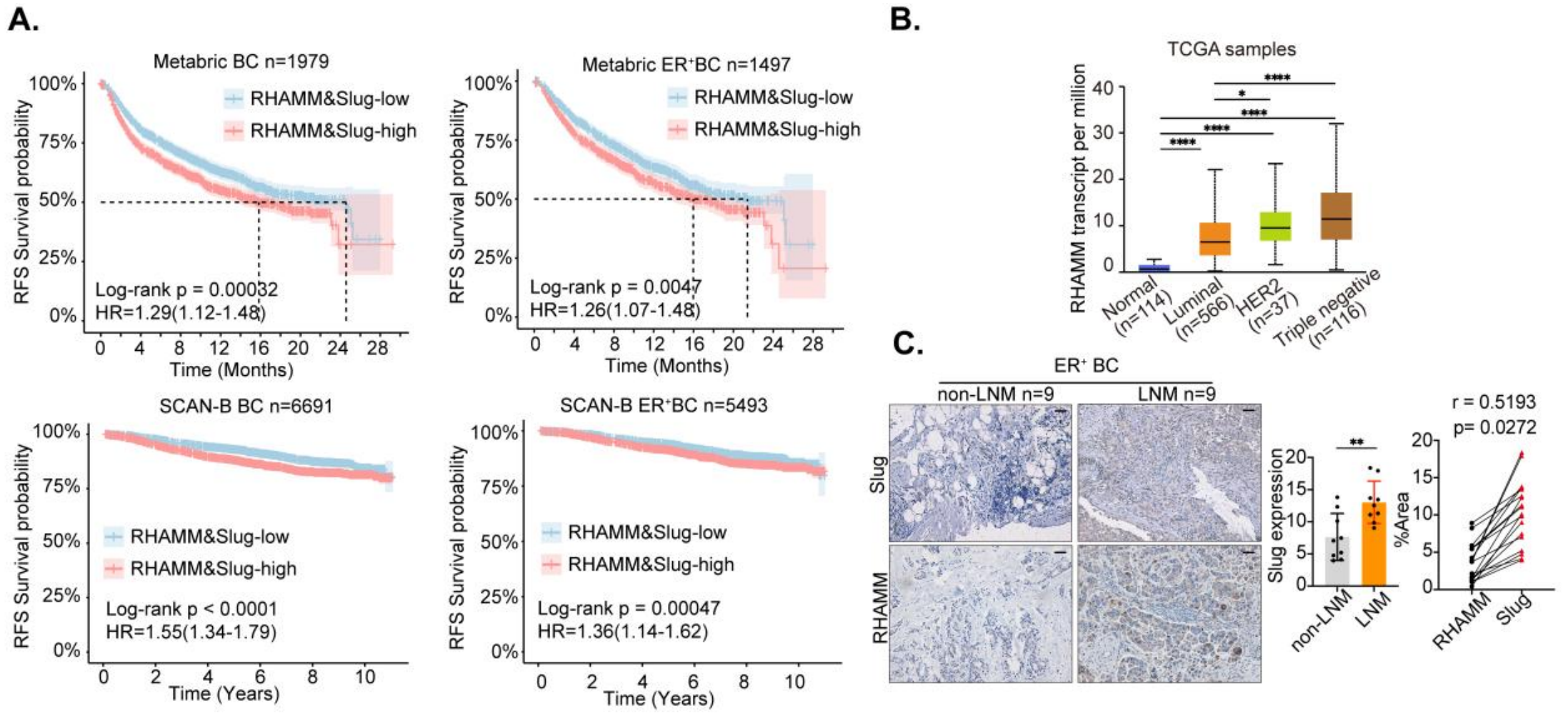

Fig. S7 Slug promotes RHAMM upregulation

(A) Kaplan-Meier analysis of recurrence-free survival (RFS) in all BC and ER$^+$ BC patients grouped by the RHAMM and Slug signature using the SCAN-B and METABRIC databases. **(B)**RHAMM expression in clinical breast cancer samples from TCGA, analyzed by UACLAN. **(C)**Representative images of Slug and RHAMM expression in ER$^+$ BC samples from patients with (LNM, n= 9) or without lympho node metastasis (non-LNM, n=9). Scale bars, 50μm. Data represent the mean (±SD). ***P* < 0.01.

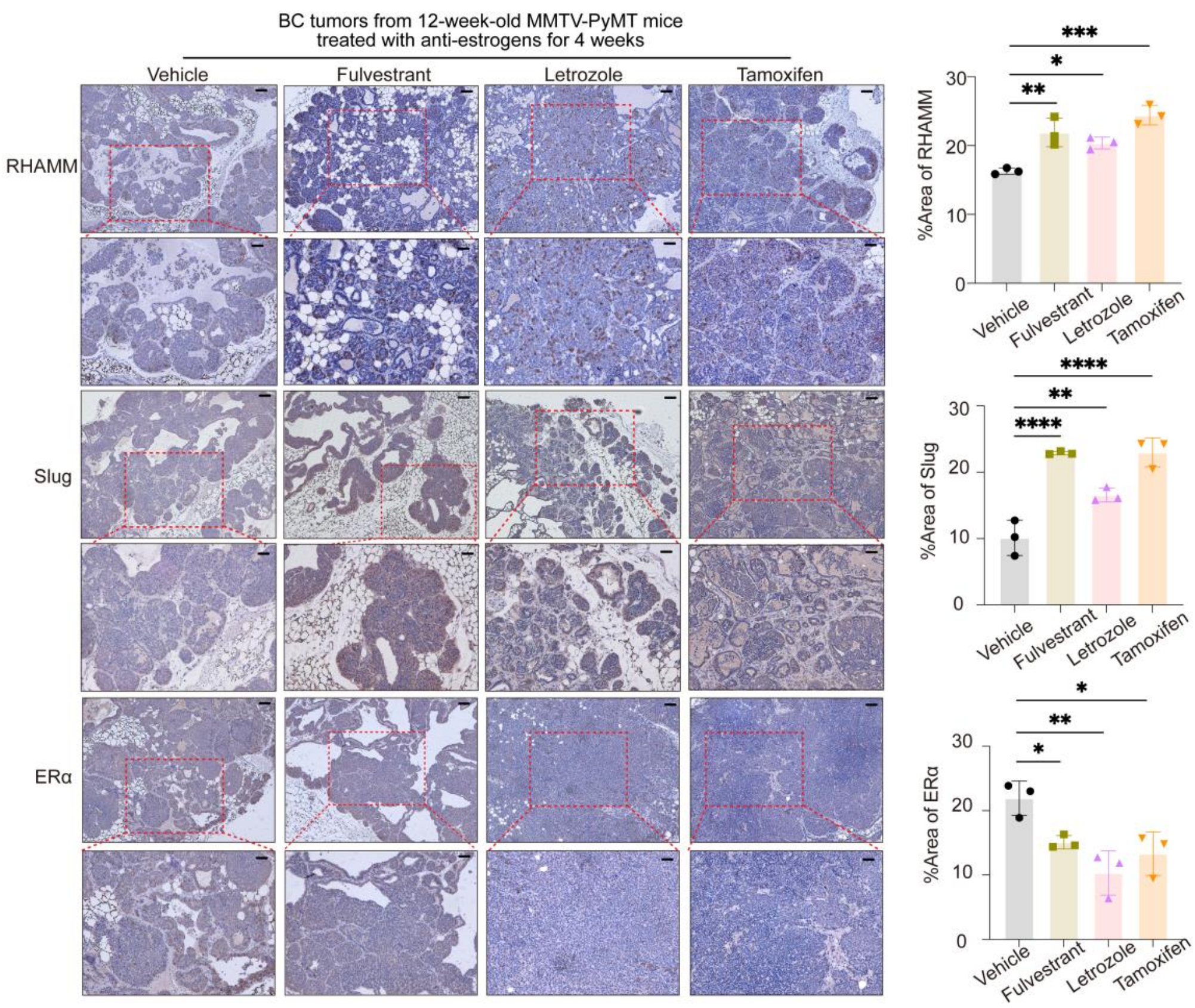

Fig. S8 ER expression profiles in vehicle- versus ER inhibitor-treated MMTV-PyMT

mammary tumors.

Representative images and quantification of RHAMM, Slug and ERα expression frequencies in primary MMTV-PyMT tumors after 4 weeks of anti-estrogen treatment. Starting at 8 weeks of age, MMTV-PyMT mice received DMSO or the following ER inhibitors: fulvestrant (50 mg/kg, s.c., weekly; Cat. No.S1191, Selleck), tamoxifen (3.3 mg/kg, p.o., 5 times/week; Cat. No. HY-13757, MCE), or letrozole (30 μg/kg, p.o., 5 times/week; Cat. No. HY-13758, MCE). The dotted lines indicate median and quartile values. Scale bar, top:100μm, bottom:50μm. *$P < 0.05$, **$P < 0.01$, ***$P < 0.001$, ****$P < 0.0001$.

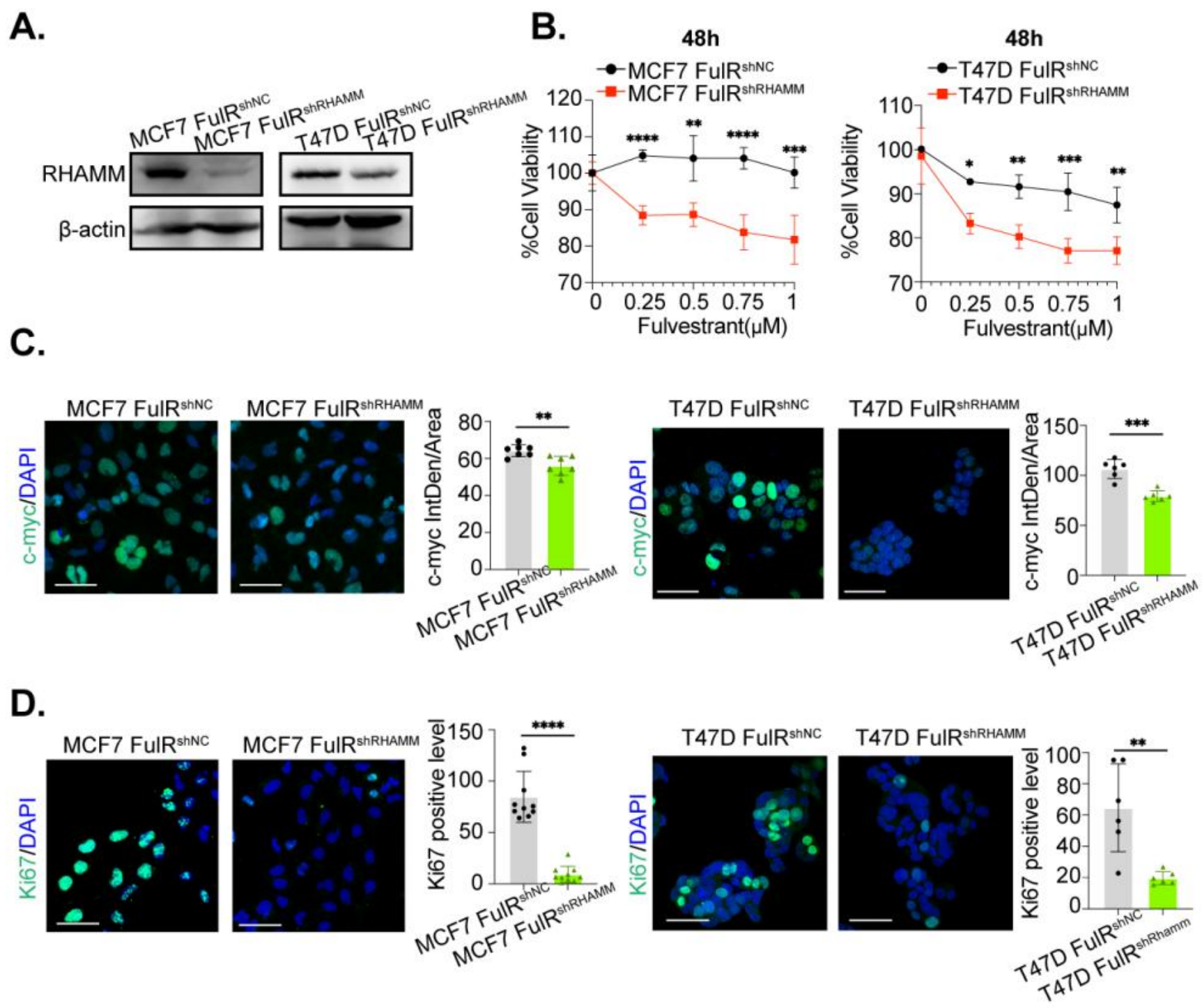

Fig. S9 Repression of RHAMM improves endocrine therapy efficiency

(A) Western blot analysis of RHAMM expression in RHAMM-knockdown fulvestrant-resistant BC cells (MCF7 FulR, T47D FulR). **(B)**CCK-8 assay was used to analyze the fulvestrant sensitivity in RHAMM-knockdown fulvestrant-resistant cell lines. **(C&D)**Immunofluorescence staining showing alterations in c-myc and Ki67 expression in RHAMM-knockdown fulvestrant-resistant BC cells. Scale bars: 50μm. Data are presented as mean (±SD) from three independent experiments. *$P < 0.05$, **$P < 0.01$, ***$P < 0.001$, ****$P < 0.0001$.

Table S1 Cell cycle contribution of all clusters of cancer cell in scRNA-seq.

	G1 counts	S counts	G2/M counts	All cells counts	G1%	S%	G2/M%
cluster6	7	469	1308	1784	0.39	26.29	73.32
cluster0	2445	1264	1831	5540	44.13	22.82	33.05
cluster1	3687	1259	514	5460	67.53	23.06	9.41
cluster2	3002	1435	581	5018	59.82	28.60	11.58
cluster3	2038	572	162	2772	73.52	20.63	5.84
cluster4	1619	631	425	2675	60.52	23.59	15.89
cluster5	938	465	567	1970	47.614	23.60	28.78
cluster7	577	188	68	833	69.27	22.57	8.16
cluster8	369	169	80	618	59.71	27.35	12.94
cluster9	158	58	38	254	62.20	22.83	14.96
cluster10	121	42	18	181	66.85	23.20	9.94

Table S2. Clinico-pathological characteristics in the studied cohort, related to Figure 2.

Tissue marker		N	Rhamm intensity of area(%) Median (25% Percentile, 75% Percentile)	*P* value
Age	<50	40	16.71(15.93, 18.14)	0.3705
	≥50	94	16.74(15.43, 18.69)	
Tumor stage	T1	54	17.42(16.23, 19.88)	0.0347
	T2	78	16.50(15.39, 17.85)	
	T3	2	19.38(17.87, 20.88)	
Nodal stage	N0	72	16.50(15.27, 17.92)	0.0126
	N1	19	17.07(16.65, 19.92)	
	N2-3	43	17.23(16.05, 19.60)	
Tumor grade	II	93	16.75(15.82, 18.10)	0.4693
	II-III	36	16.91(15.46, 19.98)	
	III	5	16.36(16.26, 16.72)	
Relapse	non-relapse	97	16.69(15.76, 18.08)	0.0178
	relapse	37	17.47(15.50, 19.96)	
	0-9%	12	16.73(16.29, 17.03)	
	10-50%	28	16.69(15.25, 17.77)	
	51-100%	16	16.98(16.29, 19.92)	

Difference between groups was tested by Mann-Whitney test or Kruskal Wallis test.

Table S3 Clinico-pathological characteristics in the studied cohort, related to Figure 5G and

Figure S7C.

Tissue marker		N	Rhamm intensity of area(%) Median(25% Percentile, 75% Percentile)	*P* value
Age	<50	8	1.78(1.36, 4.67)	0.8868
	≥50	10	2.94(1.39, 5.10)	
Tumor stage	T1	4	1.17(0.89, 1.51)	0.0772
	T2	11	3.73(1.62, 5.53)	
	T3	3	3.79(2.43, 6.35)	
Nodal stage	N0	9	1.34(1.08, 1.69)	0.0014
	N1	6	1.87(1.66, 5.39)	
	N2-3	3	5.53(4.57, 5.82)	
	10-50%	6	1.92(1.42, 4.22)	
	51-100%	2	5.23(1.55, 8.91)	

Difference between groups was tested by Mann-Whitney test or Kruskal Wallis test.

Table S4.Key resource table

Antibodies and reagents

Reagents	Source	Identifier
anti-human/mouse Rhamm	Cell Signaling Technology	87129
anti-human/mouse Rhamm	Abcam	ab124729
FITC anti-mouse CD45	Invitrogen	11-0451
PE anti-mouse EpCAM	Invitrogen	12-5791
anti-human/mouse β-actin	Servicebio	GB15001-100
anti-human CD44	Abcam	ab119348
anti-human Ki67	Invitrogen	14-5698-82
anti-human c-myc	Cell Signaling Technology	5605
anti-human ERK	Cell Signaling Technology	4695
anti-human phospho-ERK	Cell Signaling Technology	4370
anti-human MEK	Cell Signaling Technology	8727
anti-human phospho-MEK	Cell Signaling Technology	9154
anti-human JNK	Proteintech	66210-1-Ig
anti-human phospho-JNK	Proteintech	80024-1-RR
anti-human p38	Cell Signaling Technology	8727
anti-human phospho-p38	Cell Signaling Technology	4511
anti-human RAS	Cell Signaling Technology	3339
anti-human Lats1	Proteintech	17049-1-AP
anti-human Lats2	Proteintech	20276-1-AP
anti-human MST1/2	Proteintech	22245-1-AP
anti-human/mouse YAP1	Proteintech	13584-1-AP
anti-human/mouse YAP1	Santa Cruz Biotechnology	sc-101199
anti-human Septin10	Proteintech	12420-1-AP
anti-human Septin9	Proteintech	10769-1-AP

anti-human F-actin	Abcam	ab176757
anti-human p53	Cell Signaling Technology	2524
anti-human p53	Cell Signaling Technology	9282
anti-human p53	Abcam	32049
anti-human p21	Abcam	ab109199
anti-human Mdm2	Abcam	ab16895
anti-human phospho-Mdm2(Ser166)	Cell Signaling Technology	3521
anti-human/mouse Slug	Cell Signaling Technology	9585
anti-human ERα	Abcam	ab64693
Alexa Fluor® 488-conjugated goat anti-rabbit IgG	Thermo Fisher	A-11008
Alexa Fluor® 594-conjugated donkey anti-Rabbit IgG	Thermo Fisher	A-11012
Alexa Fluor® 647-conjugated donkey anti-Rabbit IgG	Thermo Fisher	A-31573
Anti-Rabbit IgG, HRP-linked Antibody	Cell Signaling Technology	7074
Anti-Mouse IgG, HRP-linked Antibody	Cell Signaling Technology	7076
Phalloidin Labeling Probes	Thermo Fisher	A22287
Alexa Fluor® 488-conjugated goat anti-mouse IgG	Thermo Fisher	A-11001
Alexa Fluor® 594-conjugated goat anti-mouse IgG	Thermo Fisher	A-11005
Alexa Fluor® 647-conjugated goat anti-mouse IgG	Thermo Fisher	A-31571
Fulvestrant	Selleck	S1191
Liberase TL	Roche	05401020001
DNAse I	Roche	11284932001
CCK-8	NCM biotech	C6005
Verteporfin	Abmole	M3539
4-methylumbelliferone(4-MU)	Sigma	M1381
Actinomycin D(Act D)	Abmole	M4881
Thymidine	Beyotime Biotechnology	ST1704
Cytochalasin D(Cyto D)	MCE	HY-N6682
Tamoxifen	MCE	HY-13757
Letrozole	MCE	HY-13758
Salirasib	MCE	HY-14754

**Deposited data**		
Deposited raw data files for RNA-seq data from this study	this paper	GSA-human: HRA020423
Deposited raw data files for	this paper	OMIX:OMIX019775

Co-IP based mass spectrometry proteomics data from this study		
Raw data files for RNA-seq	Symmans WF et al., 2010;	GEO:GSE17705;
TCGA datasets	Li T et al.,2020.	TIMER2.0
SCAN-B datasets	Saal LH et al.,2015; Brueffer C et al.,2020.	GEO:GSE60785
METABRIC transcriptomic datasets	Curtis C et al.,2012.	cBioPortal:brca_metabric

Software and algorithms

FlowJo v10Tree Star	FlowJo	https://www.flowjo.com/
R 4.4.3	R	https://www.r-project.org/
Image J 1.53.c	Image J	https://imagej.net/software/ima<br>Image J 1.53.c Image.J gej
GraphPad Prism10	GraphPad	https://www.graphpad.com/

Primer list

Site		Sequences
RHAMM	Forward	5’-GGCTGGGAAAAATGCAGAGGATG-3’
	Reverse	5’-CCTTTAGTGCTGACTTGGTCTGC-3’
ACTB	Forward	5’-CCCGAGCTGATTGACTACGG-3’
	Reverse	5’-GCAGTTCCAACGATGTCTTCG-3’
P21	Forward	5’-AGGTGGACCTGGAGACTCTCAG-3’
	Reverse	5’-TCCTCTTGGAGAAGATCAGCCG-3’
SNAI2	Forward	5’-ATCTGCGGCAAGGCGTTTTCCA-3’
	Reverse	5’-GAGCCCTCAGATTTGACCTGTC-3’

Primers for ChIP-qPCR

RHAMM promoter site		Sequences
a	Forward	5’-CGGGTCCAGATCCAAAGAGC-3’
	Reverse	5’-CCCTGCATGTTTGTGGAGTGG-3’
b	Forward	5’-TCCCTATTTTCTTTGTTGTCCTTTCC-3’
	Reverse	5’-GACAGGCAGGAAGACAACAGA-3’
c	Forward	5’-AACACGAAAATAGGCTCCTCA-3’
	Reverse	5’-AGAAAGAGGATAACACTGACCAGG-3’
P21 promoter site		Sequences
1	Forward	5’-TCTGGCCCCTCTGTGAAAAC-3’
	Reverse	5’-TATGAGCCTCCCCTCAGCAT-3’
2	Forward	5’-ATGAGCAAACTGAAAATTACTAACC-3’
	Reverse	5’-GGCCTCATCAGCAGTGGCAC-3’
3	Forward	5’-GCTTCCCAGGAACATGCTTG-3’
	Reverse	5’-AGGCAGCCCAAGGACAAAAT-3’

siRNA and shRNA

Site	Sequences
shNC	5’-TTCTCCGAACGTGTCACGT-3’
shRhamm	5’-GACCAGGACTAATGAACTA-3’
siSlug-1	5’-GAAUGUCUCUCCUGCACAATT-3’

siSlug-2	5’-CAUUAGUGAUGAAGAGGAATT-3’
siSlug-3	5’-GCUGUAAAUACUGUGACAATT-3’
siESR1	5’-UACUGUUUGCUCCUAACUUTT-3’

Supplementary Movie Legends

Movie S1. Formation of a PCC via cytokinesis failure in MCF7 FulR^{shNC} cells.

Time-lapse imaging shows a representative sequence of PCC formation in control (shNC) cells. A progenitor cell attempts mitosis but undergoes cytokinesis failure, resulting in the development of a large, multinucleated/polyploid cell. This video corresponds to the representative stages shown in Fig. 3D (top). Images were captured every 30 minutes using the FUCCI system to monitor cell cycle progression.

Movie S2. Conventional bipolar mitosis in RHAMM-knockdown MCF7 FulR cells.

Time-lapse imaging demonstrates the typical cell division pattern in RHAMM-deficient (shRHAMM) cells. Unlike the control group, these cells predominantly undergo successful bipolar mitosis, resulting in two distinct daughter cells. This video corresponds to the representative sequence shown in Fig. 3D (bottom).

Movie S3. Development and subsequent division of a rosette-like PCC in MCF7 FulR^{shNC} cells.

This video highlights the dynamic formation of a PCC through an alternative route. Following a bipolar division, a daughter cell exhibits an abnormal cell cycle, leading to the formation of a PCC with a characteristic "rosette-like" nuclear morphology. Notably, this newly formed PCC is shown undergoing a subsequent round of cell division.

Movie S4. Complex division of a PCC into multiple progeny in MCF7 FulR^{shNC} cells.

Time-lapse imaging illustrates a representative "reductive" or asymmetric division of a PCC. The large mother cell undergoes a multi-polar or irregular fission process, generating a new PCC alongside numerous smaller, viable-appearing progeny cells.

Movie S5. Abnormal multipolar division of a PCC in MCF7 FulR^{shNC} cells.

Time-lapse fluorescence imaging (related to Fig. 3H, left) reveals the aberrant mitotic machinery in a representative PCC from the shNC group. The video tracks the dynamics of Centromere B (Cenpb-EGFP, green) and α-Tubulin (mCherry, red). The PCC exhibits highly disorganized spindle assembly and irregular chromosome segregation, leading to a complex, non-canonical mitotic event. Images were captured every 3 minutes to provide high-temporal resolution of the mitotic failure and subsequent fragmentation.

Movie S6. Classical bipolar mitosis in RHAMM-knockdown MCF7 FulR cells.

Time-lapse fluorescence imaging (related to Fig. 3H, right) shows the standardized cell division process in shRHAMM cells expressing Cenpb-EGFP (green) and Tubulin-mCherry (red). RHAMM-knockdown cells demonstrate precise bipolar spindle formation and symmetrical alignment and segregation of centromeres, resulting in a successful classical mitotic division.

Supplementary Materials and Methods

Antibodies and reagents

Antibodies and reagents utilized in this study are detailed in the key resource table (*SI Appendix*, Table S4).

Mice

For animal studies, MMTV-PyMT (Fvb/NJgpt) transgenic mice, Fvb/NJgpt/WT and BALB/c nude mice were obtained from the Model Animal Research Center of Nanjing University, China. All protocols involving mice were evaluated and approved by our Institutional Animal Care and use Committee and performed under veterinary supervision.

In vivo transplants utilized fulvestrant-resistant tumors established in Passage 3 Fvb/WT mice (n=5/group) as previously reported[1]; tumors were digested, cells resuspended in PBS/2% FBS, incubated with antibodies on ice for 30 min, and analyzed by flow cytometry. $EpCAM^{+}/CD45^{-}/RHAMM^{-/+}$ cancer cell populations sorted by FACS from these tumors were orthotopically injected into mammary fat pads of 4-6 week-old female Fvb/WT mice using cold 50% Matrigel (BD Biosciences, USA); upon reaching ~100 mm^3, mice were treated with fulvestrant(50 mg/kg, intramuscular injection, weekly) and verteporfin (100 mg/kg, intraperitoneal injection, every other day) and 4MU (400 mg/kg, oral administration, daily). Tumor growth was monitored by caliper (volume = $width^2$×length ×0.52); mice were euthanized when tumor diameter reached ≥1 cm, and tumor/lung samples were excised, fixed, paraffin-embedded, and examined histologically (HE staining, IHC). For another study, female athymic BALB/c nude mice (4–6 weeks) received orthotopic injections of MCF7 $FulR^{shNC}$ or MCF7 $FulR^{shRHAMM}$ cells (8 × 10^6cells) mixed with matrigel into mammary fat pads; mice (9/group) were randomized and treated with vehicle or fulvestrant once a week.

Time-lapse cell cycle imaging

For visualizing chromosomes and cytoskeleton, cells were infected with lentiviral vectors pLenti-Cenpb-EGFP and pLenti-Tubulin-mCherry (OBiO Technology). The FUCCI system (pBOB-EF1-FastFUCCI-Puro) was a gift from Kevin Brindle & Duncan Jodrell (Addgene plasmid # 86849 ; http://n2t.net/addgene:86849 ; RRID:Addgene_86849), employing lentiviral vectors mKO2-hCdt1(30/120) and mAG-hGeminin(1/110), recorded cell cycle changes[2]. For time-lapse imaging, fluorescently labeled cells were plated on glass-bottomed six-well plates (1×10^3 cells/well) and imaged the following day using a Nikon live cell imaging system in a 37°C, 5% CO_2 humidified chamber, capturing images every 30 min for up to 4 days with a 20× objective. To estimate degradation rates of mVenus-Gem, FUCCI datasets were exported, and time courses were excised around local maxima and minima. Excised intensity data were normalised and scaled to 0-100, and fitted to the logistic growth equation below[3]: Normalised Intensity = 100/(1 + $e^{(-k(t-t1/2))}$), k is the rate of degradation of mVenus-Gem signal with a unit of 1/hour. $t_{1/2}$ is half-life

of mVenus-Gem degradation with a unit of hour, at which the signal reaches half of the maximum. Curves were fitted using lsqcurvefit function in MATLAB.

Microarray data analysis

Gene expression profiles of paired human breast tumors were downloaded from GEO (*SI Appendix*, Table S4); Log2-transformed data were collapsed to one probe per gene (GenePattern) and transformed into z-scores. Analysis of human tumor datasets was carried out as previously described[4]. Human tumor dataset analysis used the GSVA algorithm to assign enrichment scores for each sample in defined gene sets, including the RHAMM geneset (*HMMR, SNORD26, SNORD22, MIR3615, MROH7, CLEC18C, SET, FLNC, KRT6B, ANP32B, FLNA, BIRC5, RRM2, UBE2C, CENPF, PCLAF*), derived from DEGs(top5 ranked by log2FC, p-value<0.05) from cluster 6, the proteins(top5 ranked by FC) identified by co-IP/MS as interacting with RHAMM in MCF7 FulR cells, and down-regulated genes(top5 ranked by log2FC, p-value<0.05) from RHAMM-knockdown cells identified by bulk RNA-seq.

Whole-genome doubling (WGD) status, tumor purity, and ploidy for TCGA samples

TCGA samples were analysed previously using the ABSOLUTE algorithm[5], which leverage somatic copy number alterations and mutation data to estimate these parameters. WGD calls specifically integrated tumor-type ploidy distributions, homologous copy number patterns, and duplicated mutations. To compare mutational burden between WGD+ and WGD− samples, we calculated a ploidy-corrected burden (log10-transformed non-synonymous mutations/Mb divided by ABSOLUTE-inferred ploidy), assessed using Wilcoxon rank-sum tests within subtypes and linear regression (adjusting for tumor type and MSI/POLE status) across the cohort. For gene expression associations, we applied tumor type-specific linear models (RNA-seq expression ~ WGD status + ABSOLUTE purity + GISTIC2.0-derived gene-level copy number ratio [CN_Local]), with Benjamini-Hochberg FDR correction (q<0.05 significance threshold). Additionally, Kaplan-Meier curves with log-rank tests were generated to evaluate associations between WGD status and disease-free survival (DFS) in the cohort. Cross-tumor analyses averaged WGD coefficients and combined q-values using Fisher's method for visualization.

Statistical analysis

Statistical analysis used GraphPad software; normally distributed data were analyzed with unpaired two-tailed Student's t-test or paired t-test for two groups, or one-way ANOVA for multiple comparisons, ensuring similar variance between groups; nonnormally distributed data, a non-parametric Mann–Whitney test was applied. significance levels were defined as *$P < 0.05$, **$P<0.01$, ***$P<0.001$, ****$P<0.0001$

Cell culture

Human breast cancer cell lines MCF7 and T47D(STR-authenticated) were procured from the Cell Bank of the Chinese Academy of Sciences (Shanghai, China); MCF7 cells were cultured in MEMα medium(Gibco, #12571) supplemented with 10% FBS(Gibco, #10270) , 1× NEAA (Gibco, #11140), 0.01 mg/mL insulin(Sigma, #I0516) , 100 U/mL penicillin-streptomycin (Gibco, #15140), while T47D cells were cultured in RPMI-1640 medium(Gibco, #11875) supplemented with 10% FBS, 0.01 mg/mL insulin, 100 U/mL penicillin and streptomycin. The generation of fulvestrant-resistant cell lines MCF7 FulR and T47D FulR was previously described [6]. These resistant lines, generated by over-6-month exposure to 1 μM fulvestrant(Selleck, #S1191) with stepwise dose escalation, were maintained in phenol-red free media supplemented with 10% charcoal/dextran-stripped FBS (Gibco, #12676); all cell lines were confirmed negative for mycoplasma contamination(LookOut PCR Kit, negative) and Passaged at 80% confluence(split ratio 1:10) using 0.25% trypsin-EDTA (Gibco).

Single-cell data integration and analysis

The scRNA-seq data derived from previous paper[1]. It contains 6 samples from sensitive/resistant tumors. Normalization, dimensionality reduction, and clustering were accomplished with the Seurat 4.2.3 R package. The output bcl2 file was converted into FASTQ format with bcl2fastq v.2.20.0.422, and further processed with BeeNet v.1 to produce count tables. The number of barcodes in BeeNet was set to 30,000 to capture all cell-containing barcodes. Count tables were input into Seurat v.4.3.0 and merged to form a single Seurat object. Features with <300 genes, <600 unique transcript molecules, and >20% mitochondrial genes present were removed. SCTransform was used for normalization and variance stabilization. Clustering was performed in Seurat with default parameters. Differentially expressed genes were identified with the Seurat "FindMarkers" command using a Wilcoxon statistical test. Genes with adjusted $P \leq 0.05$ were considered significant.

Immunohistochemistry (IHC)

Tissues were extracted, formalin-fixed, and paraffin-embedded. Paraffin sections for immunostaining were fixed in acetone, air-dried, then dewaxed, dehydrated. Antigen retrieval performed in Tris-EDTA buffer (pH 9.0) at 95°C for 20 min using a steam heater; after PBS replenishment, sections were blocked with 5% BSA in PBS for an hour and incubated with primary antibodies(*SI Appendix*, Table S4) at 4°C overnight. Detected with goat anti-rabbit IgG-HRP (Invitrogen, #G21234, 1:500) and amplified with VECTASTAIN Elite ABC-HRP Kit (Vector Labs, PK-6100); 3,3'-Diaminobenzidine (DAB) development(Dako, K3468) provided brown staining monitored microscopically (30-90 s), stopped by immersion in distilled water. And staining intensity was evaluated using Image-J IHC Toolbox software.

Colony formation assay

Diploid and polyploid cells were isolated by FACS from Vybrant® DyeCycle™ (Thermo Fisher,

#V35003)-stained MCF7 FulR shNC and MCF7 FulR shRHAMM cells. For the colony formation assay, isolated cells were seeded at 1,000 cells per well in 6-well plates and treated with fulvestrant (1 μM, 2 μM, or 4 μM). After one week of incubation, cells were washed three times with PBS, fixed with 4% paraformaldehyde for 30 min at room temperature, and then stained with 0.1% crystal violet (BaSO Biotech Co., Ltd.) for 30 min at room temperature. Following three additional rinses, the stained colonies were imaged.

Fluorescence recovery after photobleaching (FRAP)

Fluorescence recovery after photobleaching (FRAP) followed a previous study[7], using cells stably expressing β-actin-mCherry plated in glass-bottom dishes (Corning, New York, USA); imaging was performed on a Nikon A1HD25 confocal microscope(Nikon Instruments, Tokyo, Japan), where a rectangular region was bleached after three pre-bleach frames, followed by time-lapse imaging every 16 s for 180 s using low laser power, with fluorescence intensity measured in the bleached region and recovery rate calculated as (Vt-Vpost)/(Vpre-Vpost).

Double thymidine block and cell cycle progression (flow cytometry)

MCF7 $FulR^{shNC/shRHAMM}$ cells were plated in 6-well plates and grown to approximately 30-50% confluence. Cells were subjected to a double thymidine block: incubation with 2 mM thymidine (dissolved in ddH2O) in fresh complete medium for 18 h, released by washing twice with PBS and incubating in fresh complete medium for 9 h, followed by a second incubation with 2 mM thymidine for 17 h. Following the second release from thymidine block (designated as time 0 h), cells were harvested at 0, 2, 4, 6, 8, 10, and 12 h. Harvested cells were washed with PBS, trypsinized, fixed in ice-cold 70% ethanol, and stored at -20°C for at least 24 h. Fixed cells were washed with PBS, treated with RNase A(Beyotime, China) at 37°C for 30 min, and stained with propidium iodide(PI, Beyotime, China) in PBS for 30 min at room temperature in the dark. Prior to analysis, cell suspensions were filtered through a 40 μm cell strainer. Samples were analyzed on a Beckman Coulter CytoFLEX Flow Cytometer, collecting at least 10,000 events per sample. Data analysis was performed using FlowJo v10.8.1.

Chromatin immunoprecipitation-qPCR (ChIP-qPCR) assay

Chromatin immunoprecipitation (ChIP) was performed on 10×10^5 MCF7 FulR cells crosslinked with 1% formaldehyde (Sigma, #F8775) for 10 min. Chromatin was sheared to 200–500 bp fragments using Covaris M220 (20% duty cycle, 200 cycles/burst, 180 s). Sheared chromatin was immunoprecipitated overnight at 4°C with anti-Slug antibody (Rabbit monoclonal, Cell Signaling Technology, #9585, Clone C19G7) complexed to Protein G Dynabeads™ (Thermo Fisher, #10004D). Complexes were washed, eluted, and crosslinks reversed in 200 mM NaCl with 10 μg Proteinase K (Thermo Fisher, #25530049) at 65°C overnight. DNA was purified using MinElute PCR Purification Kit (Qiagen, #28004). Quantitative PCR was performed in triplicate with TB Green™ Premix Ex Taq™ II (Takara Bio, Cat. #RR820A) on ABI 7500 Real-Time PCR System

(Applied Biosystems, USA). ChIP-qPCR fold enrichment was calculated as $2^{-(CtIP\ -\ CtInput)}$, with rabbit IgG (Cell Signaling Technology, #2729) values subtracted as background. Primers had efficiencies of 90–110%, and non-targeting genomic region primers as negative controls. Primers listed in *SI Appendix*, Table S4. Data from three independent ChIP experiments, each with triplicate qPCR reactions (mean ± SD).

siRNA and shRNA transfection

Control siRNA and siRNAs targeting human ESR1, and Slug were designed by OBiO Technology (Shanghai, China); cells were incubated in antibiotic-free medium for 24h before transfection with 100 nM siRNA using the RiboFECT™ siRNA Transfection Kit (RiboBio, Guangzhou, China) according to protocol, with knockdown efficiency assessed via Western blot or qRT-PCR (siRNA sequences in *SI Appendix*, Table S4). Lentiviral particles carrying shRNA targeting RHAMM (Lenti-shRHAMM, human) and control shRNA were also procured from OBiO Technology; both viruses were introduced into MCF7 FulR and T47D FulR cells using polybrene (5μg/ml), followed by puromycin selection for 48 hours post 24h incubation to establish stable populations.

Co-immunoprecipitation (co-IP) and mass spectrometry

Co-immunoprecipitation (co-IP): MCF7 or MCF7 FulR cells were lysed on ice in co-IP buffer [50 mM Tris-HCl pH 7.4, 150 mM NaCl, 1% NP-40, 0.5% sodium deoxycholate, 1× protease inhibitor cocktail (Beyotime, #P1048)]. Lysates (800 μg total protein) were pre-cleared with species-matched normal IgG (Rabbit IgG, Beyotime, #A7007) plus Protein A/G Magnetic Beads (50% slurry, Proteintech, #HY-K0202) at 4°C for 2 h. Supernatants were incubated with 2.5 μg anti-RHAMM antibody (Rabbit monoclonal, Cell Signaling Technology, #87129) or control IgG plus 25 μL beads overnight at 4°C. Bead-bound complexes were washed 5× with high-salt buffer [20 mM Tris-HCl pH 7.5, 500 mM NaCl, 0.1% NP-40], then eluted.

For mass spectrometry: complexes were washed with NP40 buffer, denatured in 8 M urea/100 mM Tris (pH 7.6), reduced (5 mM DTT, 37°C, 30 min), alkylated (15 mM iodoacetamide, RT, 30 min dark), digested with sequencing-grade trypsin (Promega, #V5111; 1:50 w/w, 37°C, 18 h), desalted, and analyzed on Q Exactive HF-X (Thermo Fisher) with 120-min LC gradient by Metware Technology(Wuhan, China). Data processed using MaxQuant (v2.2.0) against UniProt human database (2023_07), with peptide FDR <1% and length ≥7 aa.

CCK-8 assay

Cell proliferation was assessed using the CCK-8 assay (NCM Biotech, Shanghai, China, #C6005); 1×10^3 cells/well in 96-well plate were treated with varying fulvestrant concentrations (0.25μM - 1μM; 48 h) for specified durations, then incubated with 10 μl CCK-8 reagent(10% v/v) at 37°C for 2 h, and optical density at 450 nm was measured using a BioTek Epoch 2 microplate reader (Agilent, USA).

Immunofluorescence

For immunofluorescence, cells were fixed in 4% PFA(Sigma-Aldrich, Cat. #P6148), permeabilized with 0.25% Triton X-100(Sigma-Aldrich, Cat. #T8787), blocked with 5% BSA(Sigma-Aldrich, Cat. #A7906), incubated with primary antibodies (*SI Appendix*, Table S4) overnight at 4°C in PBS/1% BSA, followed by secondary antibodies(*SI Appendix*, Table S4) at room temperature for 1 h and DAPI(Thermo Fisher, Cat. #D1306) nuclear staining; images were captured with Nikon A1R confocal microscope (Nikon Instruments, Tokyo, Japan).

RNA sequencing

RNA sequencing was performed on RNA extracted (phenol-chloroform method) from Lenti-shRHAMM or control shRNA treated cell lines; libraries were prepared with the Illumina TruSeq RNA kit and sequenced on an Illumina HiSeq XTen system. Gene ontology and KEGG pathway analyses identified significant biological enrichments using a hypergeometric test with FDR correction ($p<0.05$).

Western blot

Total cell lysates were prepared, after BCA-based total protein normalization separated by SDS-PAGE, and transferred to PVDF membranes(Millipore, USA, Cat. #IPVH00010); membranes were blocked with 5% non-fat dry milk in TBST for 1h, incubated with primary antibodies(*SI Appendix*, Table S4) overnight at 4°C, washed by TBST(Tween-20, Sigma-Aldrich, USA), incubated with HRP-conjugated secondary antibodies(*SI Appendix*, Table S4) for 1h, and developed with ECL Plus (Thermo Fisher, USA, Cat. #32132) with signal captured by Amersham Imager 680 (Cytiva, USA). All experiments were performed in triplicate.

Quantitative real-time PCR

Quantitative real-time PCR used total RNA extracted with Trizol™ reagent (Takara Bio, Japan, Cat. #9109) from cells or tissues, cDNA synthesis was performed with 1 μg RNA using the PrimeScript™ RT Reagent Kit (Takara Bio, Cat. #RR047A). qPCR amplification was carried out with TB Green™ Premix Ex Taq™ II (Takara Bio, Cat. #RR820A) on an ABI 7500 Real-Time PCR System (Applied Biosystems, USA). Gene expression was normalized to ACTB and calculated via the $2^{-\Delta\Delta Ct}$ method.; analysis was performed in triplicate (primer sequences in *SI Appendix*, Table S4).

Dual-luciferase reporter assay

Dual-luciferase reporter assays involving cloning wild-type and mutant RHAMM promoters were designed and synthesized by Xinzhuo BioTech(Shanghai, China). Wild-type RHAMM promoter fragments(-1500 to +200 bp) were PCR-amplified from human genomic DNA and cloned into pGL4.14 via XhoI/HindIII sites. Mutants verified by sequencing and restriction digest. 293T cells plated in 96-well plates were transfected with 100 ng total DNA per well (reporter: Slug plasmid:

pRL-TK = 8:1:1 ratio) using Lipofectamine™ 3000 Transfection Reagent(Thermo Fisher, USA). Renilla luciferase activities (pRL-TK) showed <15% variation across groups and were used for normalization. Potential Slug binding sites on the RHAMM promoter were Site-1: CCACCTGC (703-710), Site-2: TGCAGGTAC(883-891), Site-3: CAGCACCTGCCCT(1055-1067), with mutagenesis primers Site-1 mut:TTCCCGAT, Site-2 mut:GATAGTCCC, Site-3 mut: TGTCATTGTAACT.

The p21 promoter(-1200 to +50 bp) in pGL3-Basic vector and p21 3′-UTR in psiCHECK™−2 vector were designed and synthesized by Xinzhuo BioTech. The p21 promoter reporter and p21 3′-UTR reporter was transiently transfected with Renilla control plasmid, in MCF7 FulR^{shNC} and MCF7 FulR^{shRHAMM} cells. Cells lysed 48 h post-transfection. Firefly/Renilla luciferase measured using Dual-Luciferase Kit (YEASEN) on SpectraMax i3x (10 s/read). Data normalized as Firefly/Renilla ratio. All experiments repeated independently ≥3 times.

Sphere formation assay and limiting dilution analysis

Sphere formation assays were conducted as described. For limiting dilution analysis, RHAMM$^{-/+}$ cancer cells were FACS-sorted (BD FACSAria III) from the fulvestrant-resistant mouse model. Cells were plated in ultralow attachment 96-well plates (Corning, #3474) at densities of 2000, 500, 100, 50, 20 cells/well using serum-free MammoCult™ Medium (StemCell Technologies, #05620) supplemented with 4 μg/mL heparin (StemCell, #07980), 0.48 μg/mL hydrocortisone (StemCell, #07925), 20 ng/mL EGF (PeproTech, #AF-100-15), 10 ng/mL bFGF (PeproTech, #100-18B) and 1 μM fulvestrant(Selleck, #S1191). After 14 days incubation at 37°C/5% CO_2 with medium replenishment every 72 h, mammospheres >50 μm diameter were counted under phase-contrast microscope (Nikon Eclipse Ti2, 20× objective; size calibrated with stage micrometer). Stem cell frequency was calculated using ELDA algorithm (http://bioinf.wehi.edu.au/software/elda/) with 10 ,000 bootstrap iterations and 95% CI. Each density plated in 8 replicates per experiment.

References

[1]LIU B, LIU Y, YANG S, et al. Enhanced desmosome assembly driven by acquired high-level desmoglein-2 promotes phenotypic plasticity and endocrine resistance in ER(+) breast cancer [J]. Cancer Lett, 2024, 600: 217179.
[2] KOH S B, MASCALCHI P, RODRIGUEZ E, et al. A quantitative FastFUCCI assay defines cell cycle dynamics at a single-cell level [J]. J Cell Sci, 2017, 130(2): 512-20.
[3]SAKAUE-SAWANO A, YO M, KOMATSU N, et al. Genetically Encoded Tools for Optical Dissection of the Mammalian Cell Cycle [J]. Mol Cell, 2017, 68(3): 626-40.e5.
[4] WANG H, LI N, LIU Q, et al. Antiandrogen treatment induces stromal cell reprogramming to promote castration resistance in prostate cancer [J]. Cancer Cell, 2023, 41(7): 1345-62.e9.
[5]QUINTON R J, DIDOMIZIO A, VITTORIA M A, et al. Whole-genome doubling confers unique genetic vulnerabilities on tumour cells [J]. Nature, 2021, 590(7846): 492-7.
[6]SUN X, TANG F, GUO Q, et al. HAS2-Ezrin-ER axis plays a role in acquired antiestrogen

resistance of ER-positive breast cancer[J]. Front Pharmacol. 2022;13:1031487.
[7]HAKALA M, WIOLAND H, TOLONEN M, et al. Twinfilin uncaps filament barbed ends to promote turnover of lamellipodial actin networks [J]. Nat Cell Biol, 2021, 23(2): 147-59.
